# Entrainment of Breast Cell Lines Results in Rhythmic Fluctuations of MicroRNAs

**DOI:** 10.3390/ijms18071499

**Published:** 2017-07-12

**Authors:** Rafael Chacolla-Huaringa, Jorge Moreno-Cuevas, Victor Trevino, Sean-Patrick Scott

**Affiliations:** 1 Grupo de Investigación en Terapia Celular y Medicina Regenerativa, Escuela de Medicina, Tecnológico de Monterrey, Monterrey NL 64710, Mexico; rafael.chacolla@cienciamed.com (R.C.-H.); jemoreno@itesm.mx (J.M.-C.); 2 Cienciamed Company, San Pedro Garza García NL 66278, Mexico; 3 Grupo de Investigación en Bioinformática, Escuela de Medicina, Tecnológico de Monterrey, Monterrey NL 64710, Mexico; vtrevino@itesm.mx

**Keywords:** circadian rhythms, rhythmic fluctuations microRNAs, microarrays, RT-qPCR, breast cancer

## Abstract

Circadian rhythms are essential for temporal (~24 h) regulation of molecular processes in diverse species. Dysregulation of circadian gene expression has been implicated in the pathogenesis of various disorders, including hypertension, diabetes, depression, and cancer. Recently, microRNAs (miRNAs) have been identified as critical modulators of gene expression post-transcriptionally, and perhaps involved in circadian clock architecture or their output functions. The aim of the present study is to explore the temporal expression of miRNAs among entrained breast cell lines. For this purpose, we evaluated the temporal (28 h) expression of 2006 miRNAs in MCF-10A, MCF-7, and MDA-MB-231 cells using microarrays after serum shock entrainment. We noted hundreds of miRNAs that exhibit rhythmic fluctuations in each breast cell line, and some of them across two or three cell lines. Afterwards, we validated the rhythmic profiles exhibited by miR-141-5p, miR-1225-5p, miR-17-5p, miR-222-5p, miR-769-3p, and miR-548ay-3p in the above cell lines, as well as in ZR-7530 and HCC-1954 using RT-qPCR. Our results show that serum shock entrainment in breast cells lines induces rhythmic fluctuations of distinct sets of miRNAs, which have the potential to be related to endogenous circadian clock, but extensive investigation is required to elucidate that connection.

## 1. Introduction

The circadian clock regulates the physiology and behavior of most species with a periodicity of approximately 24 h [1]. Mammalian cells synchronize their external and internal environments, which is critical for their well-being and survival. A lack of synchronicity might trigger diverse disorders, such as depression, diabetes, hypertension, and cancer [2].

Molecularly, the circadian system is involved in transcription, translation, protein–protein interaction, phosphorylation, and protein degradation processes, all of which participate in the biological cycles to 24 h environmental periods [3]. It is known that the circadian clock contains “clock” genes and their protein products are arranged such that they are capable of configuring an auto-regulatory feedback system [4], but the knowledge about other processes is limited. For instance, well-known clock genes such as *PER2* and *BMAL1* are expressed rhythmically in different cell types, such as adipocytes [5], myocytes [6], and stem cells [7]. They may display different phases depending on the tissue [8]. Moreover, these genes also display rhythmic expression for non-tumorigenic breast cell lines but lack of rhythmicity in tumorigenic breast cell lines (defective-clock) [9,10]. Conversely, Gutierrez et al. reported other genes displaying circadian-like expression profiles after entrainment, even in defective-clock breast cell lines [11]. This evidence suggests that additional regulatory components may be involved in the circadian system.

Recently, post-transcriptional regulatory events have been recognized as important factors in the circadian system [12]. The miRNAs are a group of short, non-coding RNAs of about 23 nucleotides that regulate the level of expression of target genes and subsequent protein translation [13]. A proteomic study of mouse liver revealed that up to 20% of soluble proteins exhibit rhythmic expression, whereas only about 10% of their transcriptional levels are rhythmic, which suggests that miRNAs may conduct a regulatory function [14]. In addition, there have been reports of particular miRNAs exhibiting rhythmic changes in expression over certain time periods in mice [15,16] and rats [17]. Thus, miRNAs seem to be a potential way in which to investigate biological timing processes that might be critical for cancer cells [18,19]. A recent study provides direct evidence that circadian disruption induces changes in miRNA levels in the mammary tissue of rats, which may lead to malignant consequences [20].

Over the last few years, there has been an increasing amount of studies linking abnormal miRNA expression to breast cancer tissue [21,22], but there is still no evidence linking periodicity, circadian clock, and miRNA expression in breast cells. Therefore, in this work, we explored a temporal expression of miRNAs among entrained breast cell lines, regardless of their circadian status (e.g., *PER2* and *BMAL1* profiles). We initiated the study by establishing cultures of breast cells, entraining with 50% horse serum, and obtaining nucleic acid samples at 4 h intervals over 48 h. Next, we analyzed the miRNA expression profiles using microarrays in three human breast cell lines, MCF-10A, MCF-7, and MDA-MB-231—over a period of 28 h. Microarray data was used to identify rhythmic miRNAs. Six miRNAs were selected to confirm their rhythmicity by reverse transcription quantitative PCR (RT-qPCR) assays over 48 h of study and testing in two additional breast cancer cell lines, ZR-7530 and HCC-1954.

## 2. Results

### 2.1. Entrainment of Human Breast Cell Cultures

**Figure 1:**
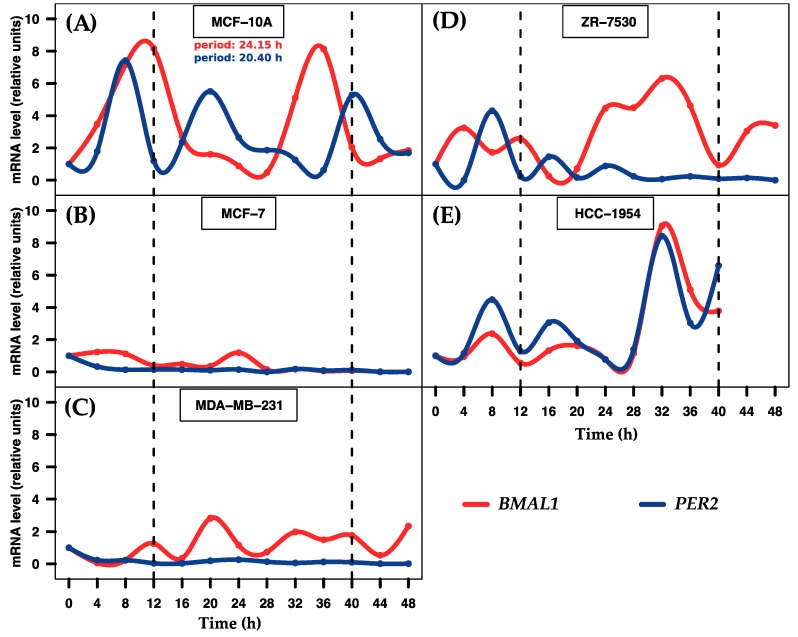
Temporal expression of *BMAL1* and *PER2* genes in five breast cell lines. The graph depicts the level of expression of two clock genes at 4 h intervals over 48 h after 2 h serum shock entrainment. (**A**) MCF-10A cells show rhythmic profiles of both genes; (**B**–**E**) MCF-7, MDA-MB-231, ZR-7530 and HCC-1954 cells do not show rhythmic profiles. Dashed black lines at 12 and 40 h were added to show the period during which the profiles exhibited robustness. Data points (means of two biological replicates) were normalized using GAPDH relative to the first time point (*t* = 0) within each corresponding cell line.

In order to analyze the temporal mRNA expression of human breast cell lines, we entrained cell cultures using the well-known serum shock method [23,24]. In order to verify the entrainment, we measured-the expression level of two known clock genes using RT-qPCR in five breast cell lines. *BMAL1* and *PER2* genes exhibited distinctive, opposite expression profiles in MCF-10A (a non-tumorigenic cell line), with periods of 24.15 and 20.40 h, respectively (see Figure 1A). Previous studies achieved similar results [9,10,11], which suggests that proper entrainment was used in our experiments. The genes did not exhibit rhythmicity in the tumorigenic cell lines—MCF-7, MDA-MB-231, HCC-1954, and ZR-75-30 (see Figure 1B–E)—as were reported previously [9,10,11]. In addition, we measured the expression level of *SERPINB1* gene in MCF-7 (see Supplementary Figure S1), which exhibited a particular rhythmic profile, as we reported previously [11]. The results confirm that MCF-10A and MCF-7 were properly entrained and support the validity of the cell culture procedures.

### 2.2. Statistical Analysis of miRNA Rhythmic Profiles

We analyzed the temporal expression (8 time points) of 2006 miRNAs in non-tumorigenic breast MCF-10A cells and two tumorigenic cell lines, MCF-7 and MDA-MB-231, using a cosine-fitting function to identify potential rhythmic miRNAs. Prior to this analysis, we generated additional data by running five types of randomization (TL (time-label), RW (row-wise), CW (column-wise), RCW (row-column-wise), and RCWB (row-column-wise by blocks)) on our experimental data (MCF-10A, MCF-7, and MDA-MB-231) to assess whether our microarray data are random. For this purpose, we assessed the distributions of the features associative to rhythmicity, including cosine correlation, period, and phase of the experimental and randomized data.

We found that the distributions of the experimental data were nearly equivalent (see Supplementary Figure S2). However, we observed differences when these distributions were compared with the distributions of randomized data. Specifically, data obtained using the TL and RW methods had similar structures to the experimental data, including similar cosine correlations, periods, and phase distributions. Data obtained using the CW and RCW methods had different structures than the experimental data, including different cosine correlations and phase distributions (but not period distributions). Data obtained using the RCWB method showed marked differences in structure compared with the experimental data, including different cosine correlations, periods, and phases (see Supplementary Figure S3A–C).

We confirmed that the RCWB method changes the structure of the experimental data. Thus, we plotted the cosine correlations and periods for experimental and randomized data. We noted that randomized data has a high proportion of transcripts with periods less than 0.22, which means they cannot be categorized as rhythmic transcript (periods close to 0.26) (see Supplementary Figure S3D). We also compared periods between the experimental and randomized data, finding that the randomized data features have fewer rhythmic periods than from the experimental data (see Supplementary Figure S3E).

In summary, the similar structures of the experimental and randomized data show that the cosine fitting function can calculate mathematical rhythmic features by chance, it is often observed when statistical analysis is performed in short temporal expression data [25]. However, our last results suggest that the RCWB method effectively removes the biological structure from experimental data and shows that the cosine fitting function can be used to calculate the circadian parameters of transcripts [26]. The current evaluation is consistent with our previous study [11].

### 2.3. Determination of miRNAs with Rhythmic Expression

**Figure 2:**
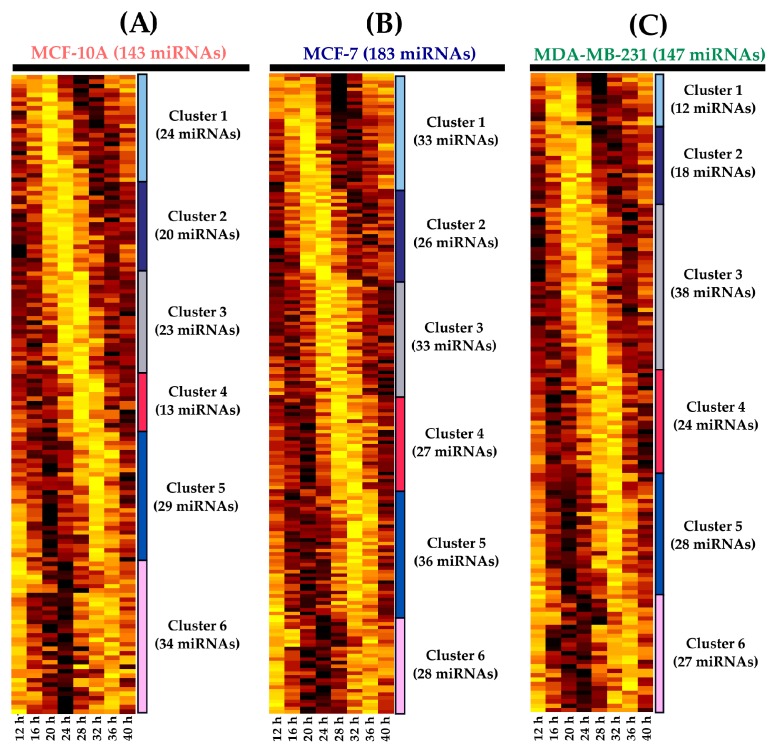

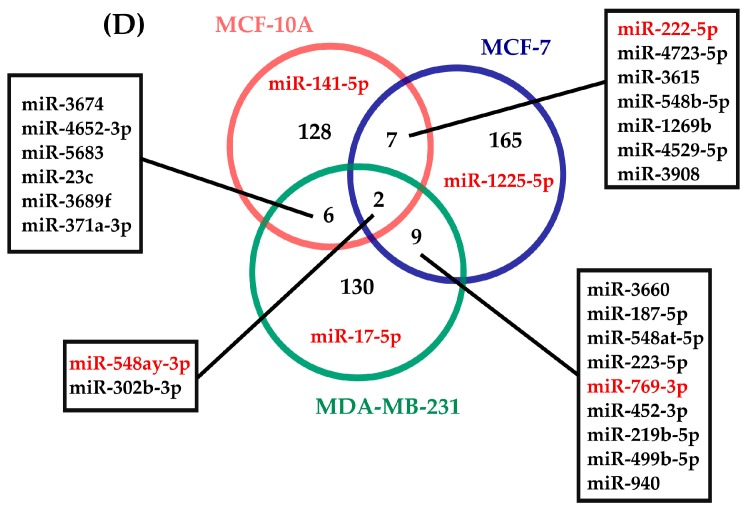
Rhythmic expression of miRNAs identified in three breast cell lines. The heat maps depict the expression level of miRNAs over 28 h (hours 12–40 of the 48 h study) in (**A**) MCF-10A; (**B**) MCF-7; and (**C**) MDA-MB-231 cells. In addition, the heat maps show 6 different clusters of miRNAs that depict marked phases. Brown colors represent higher expression values, while yellow colors represent lower expression values. Expression values are z-scaled relative to each cell line; (**D**) Venn diagram of 143, 183, and 147 miRNAs that were rhythmically expressed over 28 h in MCF-10A, MCF-7, and MDA-MB-231. The diagram also shows miRNAs that exhibited rhythmicity across cell lines and were selected for RT-qPCR validation (red).

We used the cosine-fitting function on temporal microarray data collected from MCF-10A, MCF-7, and MDA-MB-231 to identify putative miRNA with rhythmic patterns. We identified different amounts of rhythmic miRNAs in three tested breast cell lines. A total of 143 (7.12%) rhythmic miRNAs were found in MCF-10A, which can be stratified into six clusters according to phase, with apparently distinct rhythmic expression patterns (see Figure 2A and Figure S4A). MCF-7 and MDA-MB-231 had 183 (9.12%) and 147 (7.32%) rhythmic miRNAs, respectively, and their phase clusters were similar to those of MCF-10A (see Figure 2B,C and Figure S4B,C). Thus, we wondered whether the identified miRNAs were specific to certain cell lines. After our analysis, 9 additional miRNAs were found in MCF-10A and MCF-7. This observation was highly significant (*p* = 0.03, hypergeometric test). We also noted that 11 miRNAs significantly overlapped across the tumorigenic cell lines MCF-7 and MDA-MB-231 (*p* = 0.007, hypergeometric test) and 8 miRNAs overlapped across MCF-10A and MDA-MB-231 (*p* = 0.02, hypergeometric test). We identified 2 rhythmic miRNAs present in the 3 tested breast cell lines (see Figure 2D). Taken together, these diverse results show a large component of specific rhythmic miRNAs and only small common component feature rhythmicity among the tested breast cell lines. This is presented in Tables S1–S3, which depict the miRNAs, cosine correlations, periods, and phases obtained from the cosine-fitting function.

### 2.4. Validation of Rhythmic Expression Profiles Using RT-qPCR

For validation purposes, we selected miRNAs with similar or opposite rhythmic profiles. We selected one miRNA that displayed rhythmicity only for MCF-10A (miR-141-5p), MCF-7 (miR-1225-5p), and MDA-MB-231 (miR-17-5p). Additionally, we selected miR-769-3p because it shared a similar profile for MCF7 and MB-MDA-231 and miR-222-5p because it had opposite profiles for MCF-10A and MCF-7, according to the microarray data. Finally, we included miR-548ay-3p because it exhibited rhythmic expression in MCF-10A, MCF-7, and MDA-MB-231 (see Figure 2D). In order to provide a broader view of the rhythmic expression of miRNAs, we validated the expression using RT-qPCR assays over 48 h. Additionally, we measured the temporal expression of the selected miRNAs in two other tumorigenic breast cell lines, ZR-7530 and HCC-1954 (note that the last two time points of HCC-1954 are missing).

**Figure 3:**
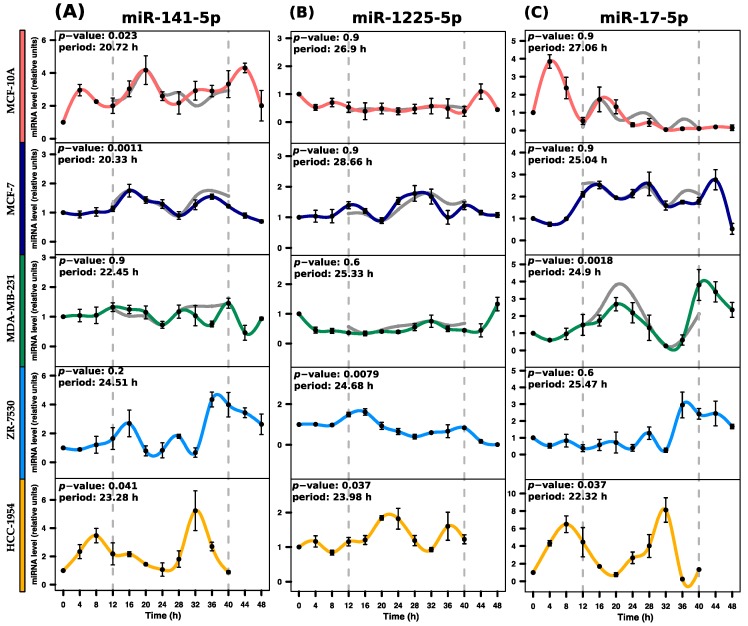
Expression profiles of miR-141-5p, miR-1225-5p, and miR-17-5p across breast cell lines as determined by RT-qPCR. Their rhythmic expression was evaluated by RT-qPCR in serum-shocked MCF-10A, MCF-7, MDA-MB-231, ZR-7530, and HCC-1954 cells over 48 h (4 h intervals). (**A**) miR-141-5p exhibits rhythmic profiles in MCF-10A, MCF-7 and HCC-1954 cells; (**B**) miR-1225-5p exhibits rhythmic profiles in ZR-7530 and HCC-1954 cells; (**C**) miR-17-5p exhibits rhythmic profiles in MDA-MB-231 and HCC-1954 cells. Data points (means of three biological replicates ± standard error of the mean (SEM)) were normalized using miR-106a-5p relative to the first time point (*t* = 0). The *p*-value and period values of rhythmic profiles were obtained from the MetaCycle analysis and are illustrated at the top of each plot. Expression profiles with *p*-values less than 0.05 were considered rhythmic. For comparison purposes, the gray lines in the graphs concerning MCF-10A, MCF-7, and MDA-MB-231 illustrate the expression profiles obtained from the microarray data, which were scaled to the expression profile obtained from RT-qPCR assays. Dashed gray lines at 12 h and 40 h were added to show the period in which profiles were measured by microarray.

**Figure 4:**
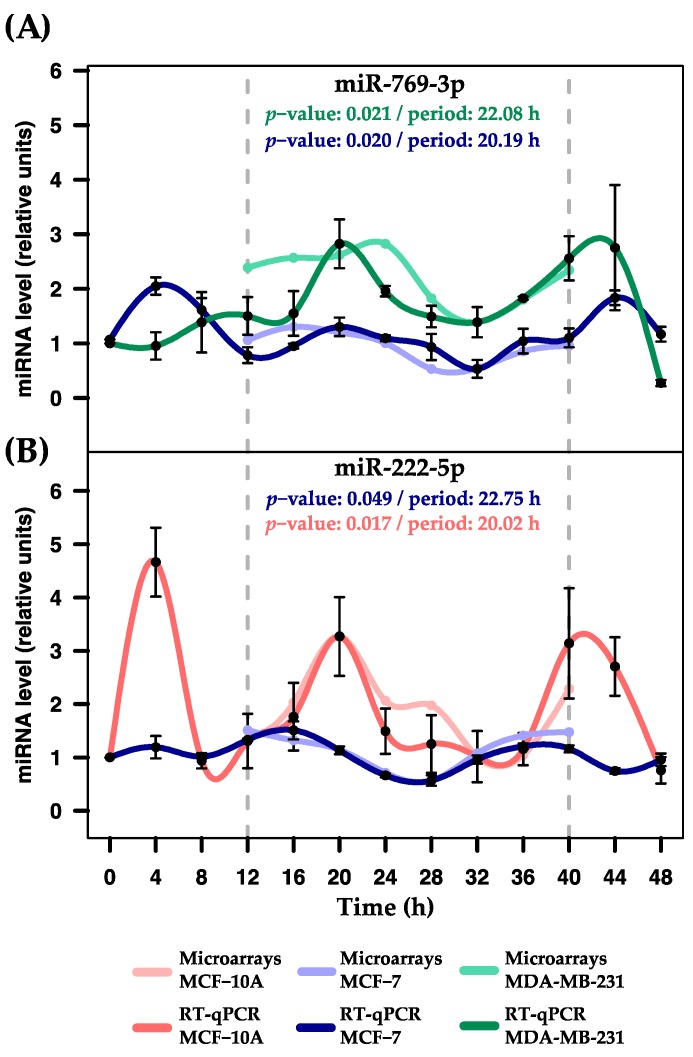
Expression profiles of miR-769-3p and miR-222-5p in three breast cell lines. Rhythmic expression was evaluated using RT-qPCR in serum-shocked MCF-10A, MCF-7, and MDA-MB-231 cells over 48 h (4 h intervals). (**A**) Rhythmic profiles of miR-769-3p obtained from microarray and RT-qPCR assays in MCF-7 and MDA-MB-231 cells; (**B**) Rhythmic profiles of miR-222-5p obtained from microarray and RT-qPCR assays in MCF-10A and MCF-7 cells. Data points (means of three biological replicates ± SEM) were normalized using miR-106a-5p relative to the first time point (*t* = 0). The *p*-values and period values of rhythmic profiles were obtained from MetaCycle analysis and are illustrated at the top of each plot. Expression profiles with *p*-values less than 0.05 were considered rhythmic. For comparison purposes, the more lightly colored lines illustrate the expression profiles obtained from microarray assays, which were scaled to the expression profiles obtained from RT-qPCR assays. Dashed gray lines at 12 and 40 h were added to show the periods in which the profiles were measured by microarrays.

**Figure 5:**
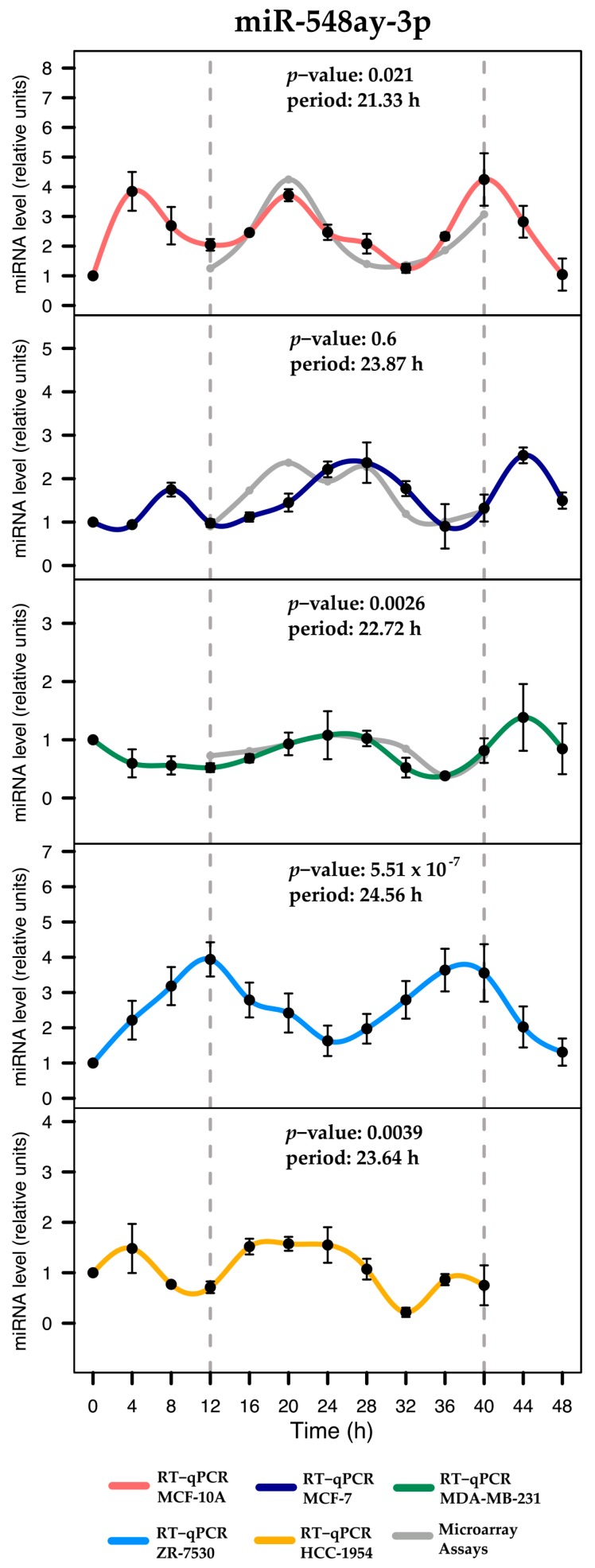
Expression profiles of miR-548ay-3p across breast cell lines. Rhythmic expression was evaluated using RT-qPCR in serum-shocked MCF-10A, MCF-7, MDA-MB-231, ZR-7530, and HCC-1954 cells over 48 h (4 h intervals). Data points (means of three biological replicates ± SEM) were normalized using miR-106a-5p relative to the first time point (*t* = 0). The *p*-values and period values of rhythmic profiles were obtained from MetaCycle analysis and are illustrated at the top of each plot. For comparison purposes, the gray line illustrates the expression profiles obtained from microarray assays, which were scaled to the expression profiles obtained from RT-qPCR assays. Dashed gray lines at 12 and 40 h were added to show the period in which the profiles were measured by microarrays.

After comparing the miRNAs temporal expression between microarray and RT-qPCR results, we observed similar profiles, suggesting reproducibility among assays (see Figure 3, Figure 4 and Figure 5). The significance of the rhythmicity of miRNAs was evaluated using MetaCycle [27] (*p* < 0.05). In MCF-10A, miR-141-5p displayed the expected rhythmicity (MetaCycle *p*-value = 0.023). However, this miRNA also displayed significant rhythmicity in MCF-7 and HCC-1954 (MetaCycle *p*-values of 0.0011 and 0.041, respectively), which was not observed in the microarray data. We observed that miR-141-5p exhibited lower amplitude in MCF-7 than MCF-10A and HCC-1954 (see Figure 3A). As observed in the microarray analysis, miR-1225-5p did not display significant rhythmicity in MCF-7 cells, but it did display rhythmicity in ZR-7530 and HCC-1954 (MetaCycle *p*-values of 0.0079 and 0.037, respectively). Nonetheless, it exhibited low amplitudes across five breast cell lines (see Figure 3B). The miR-17-5p displayed the expected rhythmicity in MDA-MB-231 cells (MetaCycle *p*-value = 0.0018), and it also exhibited rhythmicity in HCC-1954 (MetaCycle *p*-value = 0.037) (see Figure 3C).

The miR-769-3p displayed significant rhythmicity in MCF-7 and MDA-MB-231, both of which featured MetaCycle *p*-values close to 0.02 and demonstrated profiles consistent with those obtained from the microarray (see Figure 4A). The miR-222-5p displayed significant rhythmic expression in MCF-10A and MCF-7 (MetaCycle *p*-values of 0.017 and 0.049, respectively) and profiles consistent with the microarray results (see Figure 4B). These miRNAs were also evaluated in the remaining three breast cell lines and demonstrated non-significant rhythmicity (data not shown). The miR-548ay-3p exhibited the expected rhythmic expression in MCF-10A and MDA-MB-231 cells (MetaCycle *p*-value = 0.021 and 0.0026, respectively), but not in MCF-7 cells (Figure 5). However, it displayed significant rhythmicity in ZR-7530 and HCC-1954 (MetaCycle *p*-values of 5.51 × 10^−7^ and 0.0039, respectively). Additionally, we observed that mir-548ay-3p exhibited large-scale peaks of expression in MCF-10A and ZR-7530, with, interestingly, opposite profiles (Figure 5). See Table 1 for the rhythmic features and statistical significance of the six miRNAs determined by validation.

### 2.5. Identification of the Target mRNAs in a Group of miRNAs

After the six miRNAs were validated, we sought their gene targets in multiMiR [28]. It lists the gene targets that were experimentally validated for each miRNA. Of the six miRNAs, only miR-17-5p revealed targets (343 genes in total). We then performed an enrichment analysis using the Reactome enrichment tool, which identified 200 enriched pathways (see Supplementary Table S4). We observed the circadian clock pathways (*p* = 0.0027, FDR = 0.049, rank = 51) of five genes, MEF2D, PPP1CA, NPAS2, PER1, and RPS27A.

## 3. Discussion

The circadian system is responsible for cell-signaling processes during periods of about 24 h whose disruption might be associated with transcriptional and post-transcriptional changes in normal and tumorigenic tissues [29]. In this system, BMAL1 and PER2 proteins play a critical role in the functioning of the molecular clock. BMAL1 (also known as ARNTL) and its partner CLOCK comprise a core transcription complex that is needed to generate circadian oscillations in cells [30]. PER2 directly and rhythmically binds to this complex to drive the circadian negative feedback loop [31]. These components are necessary for regulation of circadian rhythms within individual non-tumorigenic cells.

To further understand the circadian clock at the molecular level, the scientific community has begun to explore the non-coding class of small RNAs known as miRNAs, which regulate gene expression [32]. There is evidence that some miRNAs display rhythmic changes in expression over certain periods in mice and rats [15,16,17]. Hence, we sought to explore the expression changes of miRNAs by a 48 h time-course study after serum shock entrainment of human non-tumorigenic and tumorigenic breast cell lines regardless *BMAL* and *PER2* status. To determine if there are miRNAs that may exhibit rhythmic fluctuation across breast cell lines, we first entrained the cell cultures and measured the temporal expression of *BMAL1* and *PER2* genes to assess the status of the circadian system. These genes displayed the circadian characteristic expression profiles in MCF-10A (non-tumorigenic), but not in the remaining breast cell lines assayed, as previously reported [9,10,11]. Moreover, the association between disrupted circadian rhythm and cancer is well known [33]. Despite this, we have previously reported some coding genes expressed rhythmically in clock-defective breast cell lines [11]. We confirmed the pattern for SERPINB1 in MCF-7 cells [11]. This evidence suggests that may exist alternative mechanisms that induce rhythmic, but further investigation is required to elucidate this hypothesis.

Diverse post-transcriptional mechanisms have been reported to regulate the circadian clock in mammalian cells [12]. The regulatory function of miRNAs, especially regarding circadian expression, was observed in clock genes [34] and the liver transcriptome [18] of mammalian cells. However, it is still unknown if miRNAs display a rhythmicity feature in non-tumorigenic and tumorigenic cell lines. Prior to identifying these miRNAs, we confirmed that our data results are not random by contrasting the distributions of the rhythmic features calculated from experimental data with those in data without a biological structure (patternless and randomized). The RCWB method changed noticeably the structure of the experimental data. The results can be explained by the simultaneous shuffling of columns and rows in two separate blocks to ensure a lack of intrinsic pattern in the data [26]. Biologically, it might be justified by the assumption that miRNAs feature diverse temporal patterns of expression [35], such as starting and ending expressions with slow peaks or dampened expression. Therefore, our results suggest that short time course data should be randomized using an appropriate method, such as RCWB, to achieve more accurate null distributions [26]. We believe that our validation of miRNA profiles using RT-qPCR is the best evidence that microarray data are not random.

Rhythmic miRNAs were classified based on cosine correlation, period, amplitude, and phase reaching a total of 143, 183, and 147 miRNAs identified for MCF-10A, MCF-7, and MD-MBA-231, respectively. These results showed that less than 10% of miRNAs maintained rhythmicity across distinct breast cell lines, consistent with the hypothesis that the expression of miRNAs depends on the origin of the breast cells [36]. Notably, some rhythmic miRNAs display distinct phases across cell lines (observed in Figure 3, Figure 4 and Figure 5). Phase changes have been also observed in clock genes in liver and kidney tissues from mice with colorectal liver metastases [37]. Although miRNAs in these conditions have not yet been explored, our observations suggest that shifts in phases are common depending on the cell type origin.

Our analysis identified 9 miRNAs that exhibit rhythmicity in both MCF-10A and MCF-7, but at different phases. This suggests that both cell lines have distinct internal timing in biological processes, likely due to their estrogen receptor (ER) status, which is over-expressed in MCF-7 cells and involved in circadian molecular machinery [38]. In addition, 8 miRNAs were determined to exhibit rhythmicity in both MCF-10A and MDA-MB-231 also with shifts in phases, nevertheless estrogen receptor is unlikely to be involved in MDA-MB-231 due to its feature of low ER expression. Moreover, 11 miRNAs found in both MCF-7 and MDA-MB-231 also exhibited shifts in phases. This may be due to their subtype of breast cancer or ER status, MCF-7 (ER(+) and PR(+)), while MDA-MB-231 (ER(−), PR(−), and HER2/neu(−)).

Next, we validated the rhythmic expression of six miRNAs that were present in one or more cell lines using RT-qPCR across five breast cell lines. Overall, we observed that the non-validated miRNAs profiles were mostly associated with short amplitudes in the microarray results. This issue often arises in validation assays [39]. Apparently, the short amplitudes may be noise instead of a rhythmic signal. From the miRNAs that exhibited rhythmicity in the microarray results, the miR-141-5p that exhibited rhythmicity only for MCF-10A was also detected in MCF-7 and HCC-1954 by RT-qPCR. The fact that these three breast cell lines with distinct features share this miRNA rhythmicity is intriguing and should be further studied. Notably, one study reported that miR-141-5p showed circadian expression in rat enterocytes [17]. We also found that miR-1225-5p, which was observed in the microarray analysis in MCF-7 was not validated by RT-qPCR, but surprisingly, the profile of miR-1225-5p seems rhythmic in ZR-7530 and HCC-1954 cells although at low amplitudes, suggesting randomness. The miR-17-5p exhibited rhythmic expression in MDA-MB-231 and HCC-1954. Interestingly, both cell lines are negative for ER and PR, but differ in terms of ERBB2 (HER2/neu) expression. Thus, the rhythmicity exhibited in both cell lines appears to be beyond the biological function of the receptor tyrosine-kinase. Additionally, this miRNA is a member of the well-known miR-17/92 cluster, which is largely related to breast cancer as an oncogene and tumor suppressor [40,41]. We also noted that miR-17-5p has potential 343 targeted mRNAs, five genes of which (*MEF2D*, *PPP1CA*, *NPAS2*, *PER1*, and *RPS27A*) are involved in the circadian clock pathway (*p* = 0.0027). Interestingly, a recent study found that miR-17-5p stabilizes the circadian clock period by inhibiting the translation of Clock and Npas2 in mice [42]. These results are encouraging and propose that the other miRNAs identified in the same way than mir-17-5p may play a potential role in rhythmicity. Nevertheless, further investigation is required to validate the remaining molecular associations.

Additionally, we validated the microarray results identifying miRNAs that exhibit shared rhythmicity among MCF-10A, MCF-7, and MDA-MB-231. The miR-769-3p displayed similar rhythmic profiles for MCF-7 and MDA-MB-231. However, the temporal expression of this miRNA for MCF-7 exhibited lower amplitude. These results agrees with evidence showing that miRNA biogenesis is altered in cells with ERα over-expression [43], while MDA-MB-231 are ERα-negative cells that presumably have different miRNA expression. Taken together, this evidence suggests that both cell lines share some inner mechanisms, despite the fact that they are distinct subtypes of breast cancer. The miR-222-5p displayed rhythmicity in MCF-10A and MCF-7 with markedly distinct amplitudes. Previous studies reported that miR-222-5p plays a role in the regulation of ERα expression in breast cancer cells by promoting the transition from ER-positive to ER-negative tumors during the progression of cancer [44] and related to circadian clock outputs [45]. Taken together, this evidence suggests that miR-222-5p expression may participate in different mechanisms between both breast cell lines unknown as of yet. The rhythmic expression of miR-548ay-3p was validated in MCF-10A and MDA-MB-231, but not in MCF-7. Interestingly, it also exhibited rhythmic patterns in ZR-7533 and HCC-1954. To date, no studies have investigated this miRNA because it was discovered only recently [46].

**Figure 6:**
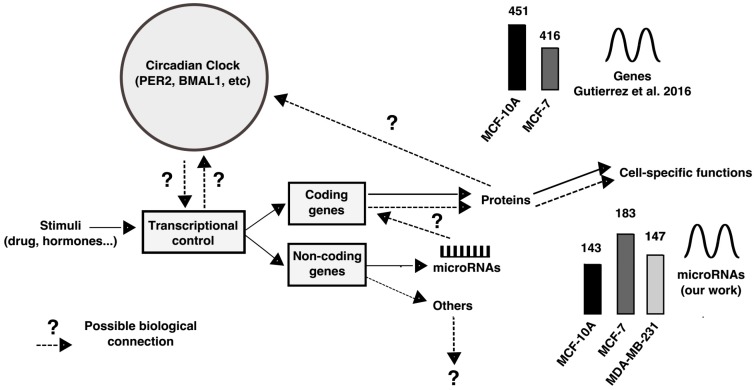
Overview of circadian control and the insight of the rhythmic miRNAs in this system. Continuous lines between concepts represent known facts. Dashed lines represent unknown information. Particularly, derived from our study it is still unknown what could be the role of rhythmic miRNAs to specific cell function and to circadian control. Circadian control inspired in [47].

In general, stimuli (e.g., drugs, hormones)—in this case serum shock—might exert transcriptional control (induction) over coding and non-coding genes (see Figure 6), which further play roles in specific cell functions or as a feedback loop in the circadian system. For coding genes, the mechanisms are well known [47]*.* In breast cell lines, we have previously observed similar number of rhythmic coding genes but at different amplitudes [11]. In this work, we have shown the miRNA may also be rhythmic. It is unknown how this emerges, what is their contribution to tune specific cell functions, or their possible role in the feedback loop. Our data may contribute in this direction.

## 4. Materials and Methods

### 4.1. Cell Lines and Culture Procedures

The breast cell lines MCF-10A (ATCC^®^ CRL­10317™), MCF-7 (ATCC^®^ HTB­22™), and HCC-1954 (ATCC^®^ CRL­2338™) were purchased from the American Type Culture Collection (ATCC^®^, Rockville, MD, USA). MDA-MB-231 and ZR-75-30 cell lines were donated by Nadia Jacobo Herrera from Instituto Nacional de Ciencias Médicas y Nutrición “Salvador Zubirán” in Mexico City, Mexico. Non-tumorigenic human breast cells (MCF-10A) were grown in Mammary Epithelial Basal Medium^®^ supplemented with SingleQuots^®^ from the MEGM^®^ BulletKit^®^ (Lonza, Walkersville, MD, USA). The tumorigenic breast cell lines, ZR-75-30 and HCC-1954, were grown in Gibco^®^ Roswell Park Memorial Institute (RPMI)-1640 medium (Thermo Fisher Scientific, Grand Island, NY, USA) supplemented with 10% and 15% heat-inactivated Gibco^®^ Fetal Bovine Serum (FBS) (Thermo Fisher Scientific), respectively. The MCF-7 and MDA-MB-231 cell lines were grown in Gibco^®^ Dulbecco’s Modified Eagle Medium, which features a nutrient mixture of D-MEM and F-12 (Thermo Fisher Scientific), supplemented with 10% heat-inactivated FBS. Each culture medium was supplemented with Gibco^®^ Penicillin-Streptomycin antibiotics (Thermo Fisher Scientific). Cell cultures were grown at 37 °C with 5% CO_2_ and 95% air in a humidified incubator.

### 4.2. Serum Shock Synchronization

Cells were seeded in six-well plates and until they reached near-confluence. Then, cells were synchronized using the serum shock procedure [10]. One day before serum shock, the plates with confluent cells were washed and left overnight in serum-free basal medium (starvation). RPMI-1640 was the basal medium for the ZR-75-30 and HCC-1954 cells, while D-MEM/F-12 was used for the MCF-10A, MCF-7, and MDA-MB-231 cells. After overnight starvation, the medium was exchanged with the appropriate basal medium, which contained 50% horse serum (Biowest, Mountain View, CA, USA). After incubation for 2 h at 37 °C, the medium was replaced with a serum-free basal medium. At this time (time zero), we started to harvest cells every 4 h for 48 h. Time samples were collected separately to confirm synchronization (by duplicate) and miRNA expression (by triplicate; two wells of a six-well plate were merged to make one replicate). This protocol was performed for all breast cell lines.

### 4.3. Total RNA Purification for mRNA and miRNA Expression

Time samples obtained to confirm synchronization were directly lysed by 400 μL of TRI Reagent^®^ (Sigma-Aldrich, St. Louis, MO, USA) to achieve total RNA isolation according to the manufacturer’s instructions. The total RNA was purified using 3 M sodium acetate, with a pH of 5.2 (1/10 volume), and 2.5 volumes of cold 100% ethanol. The mixture was incubated at −80 °C for 30 min. Next, the mixture was centrifuged at 13,500× *g* for 30 min, washed with 1 mL of 75% ethanol, and then dried and re-suspended in pre-warmed nuclease-free water. We then measured the yield and purity of the purified total RNA using NanoDrop 1000 (Thermo Fisher Scientific).

Time samples obtained for miRNA expression were disassociated with 0.4% Trypsin-EDTA (BioVision, Milpitas, CA, USA) and washed with basal medium 2–3 times. Then, the cell pellets were immediately processed to collect cytosolic fractions using the Nuclear/Cytosol Extraction Kit (BioVision) according to the manufacturer’s instructions. These fractions were stored at −70 °C. Next, we isolated total RNA from cytosolic time fractions using the MIRVANA miRNA Ambion^®^ Kit (Thermo Fisher Scientific) according to the manufacturer’s instructions. The RNA from cytosolic fractions (cyRNA) was purified using 3 M sodium acetate at a pH of 5.2 (1/10 volume) and 2.5 volumes of cold 100% ethanol. Then, the mixture was incubated at −70 °C for 30 min. Next, the mixture was centrifuged at 13,500× *g* for 30 min, washed with 1 mL of 70% ethanol, dried, and re-suspended in pre-warmed nuclease-free water. We then measured the yield and purity of the cyRNA using NanoDrop 1000 (Thermo Fisher Scientific). In addition, cyRNA samples were assessed using an RNA HighSense Analysis Kit (Bio-Rad Laboratories, Irvine, CA, USA) in Experion (Bio-Rad Laboratories) in order to ensure the quality of RNA (RIN, RNA integrity number) for microarray hybridization assays.

### 4.4. Quantitative RT-qPCR for mRNA Expression

Reverse transcription of mRNA to cDNA was carried out with 400 ng of total RNA using an AffinityScript Multi-Temperature cDNA synthesis kit (Agilent Technologies, Santa Clara, CA, USA) according to the manufacturer’s instructions. Next, qPCR was carried out using the Applied Biosystems^®^ StepOne™ Real-Time PCR System (Thermo Fisher Scientific). The reaction involved 20 ng of cDNA, 200 nM of forward and reverse primers for circadian clock genes, *BMAL1*, and *PER2*, and one rhythmic gene for MCF-7, SERPINB1, which we described in a previous study [11] (Table 2), 1× Brilliant II Fast SYBR^®^ Green qPCR Master Mix (Agilent Technologies), 300 nM passive reference dye, and molecular grade water to reach a final volume of 12 µL. The initial activation step was performed at 95 °C for 3 m, then a cycling program was begun, which involved denaturing at 95 °C for 6 s and annealing/extension at 60 °C for 12 s, and a melting curve analysis was performed to ensure the efficiency of the reaction. Relative expression was calculated using the ΔΔ*C*_t_ method [48] with GAPDH serving as the reference gene for normalization. The expression level of each biological replicate was calculated separately and averaged, and the SEM was calculated for each time point. The relative expression results of each gene were plotted over 48 h at 4 h intervals.

### 4.5. Microarray Processing and Analysis

The miRNAs’ temporal expression was profiled using a Human miRNA Microarray 8 × 60 K Kit (Agilent Technologies), which contained 2006 human miRNAs, based on miRBase Release 19.0 (http://www.mirbase.org/). These assays were performed using cyRNA time samples during a period of 28 h (hours 12–40 of the 48 h study) because they show the beginning of gene-sustained oscillations in living cells upon serum shock [23,49]. Thus, we used two slides (eight arrays per slide) for to profiling MCF-10A, MCF-7, and MDA-MB-231.

In total, 100 ng of cyRNA time samples was used for hybridization with miRNA complete labeling and the Hyb Kit (Agilent Technologies) according to the manufacturer’s instructions. The first slide was hybridized with cyRNA time samples from MCF-10A labeled with Cy3 fluorochrome, while cyRNA time samples from MCF-7 and MDA-MB-231 were labeled with Cy3 and pCp (Cytidine-5′-phosphate-3′-(6-aminohexyl)phosphate)-Cy5 (277 µM) fluorochromes, respectively, and hybridized with the second slide, according to the manufacturer’s instructions [50]. Next, standard washing procedures and hybridization with microarray slides were performed according to the manufacturer’s instructions. Slides were scanned using the GenePix^®^ 4000B Microarray Scanner (Molecular Devices, Downingtown, PA, USA) with GenePix^®^ Pro 6.0.1.25 Acquisition and Analysis Software (Molecular Devices). The GenePix^®^ results (GPR) files were imported into the software R, version 2.12.2 (http://www.R-project.org). Spot intensities were background subtracted using the Linear Models for Microarray Data package (Limma, version 3.20.5, https://bioconductor.org/packages/limma/) [51,52]. The miRNA array contained an average of 30 replicates per probe sequence, with a total of 60,000 unique features and controls. The data were normalized between arrays by quantile normalization and log10 transformed, and the median was applied to the replicated probes [53]. The miRNA expression data contains one to four different probes associated with each miRNA, which were considered individually for further analysis. However, miRNAs were selected for validation based on restrictive parameters, as described in Section 4.6. The miRNA microarray data will be submitted to GEO NCBI.

### 4.6. Identification of miRNAs with Rhythmic Expression

The miRNAs with rhythmic patterns were identified by analyzing miRNA expression over 28 h (hours 12–40 of the 48 h study) for MCF-10A, MCF-7, and MDA-MB-231. Firstly, data was arranged from 0–28 h in order to perform the cosine-fitting algorithm. This interval, which avoids the first 12 h, is appropriate for identifying miRNAs with robust rhythmicity [49]. The cosine function is defined as *g = β* × cos(*ω × t + ϕ*), where the cosine parameters β, ω, and ϕ were limited to [0, 30], [0, π], and [−2π, 2π], respectively. The cosine fitting function was evaluated by comparing the distributions of the cosine correlations, periods, and phases in the experimental and randomized data. Afterward, restrictive values for amplitude, period, and cosine correlation were selected to identify rhythmic miRNAs. These values were *r* > 0.82 for cosine correlation, 0.22–0.29 for period, and >0.045 for amplitude. The miRNAs that fulfilled those parameters were grouped using hierarchical clustering (ward agglomeration) to identify miRNAs with similar phases. Next, miRNAs were selected for confirmation using RT-qPCR. They were chosen if they featured cosine-fitting correlation values of *r* were close to 1, a period of about 24 h, and a high amplitude value. Additionally, we restricted the selection of miRNAs for longer probe sequences, lengths of at least 15 nt, and/or high correlation among probes that belong to the selected miRNA. These parameters were used to ensure the reproducibility of the RT-qPCR assays [54]. Finally, the newly developed MetaCycle package (version 1.0.0; https://github.com/gangwug/MetaCycleV100.git) was used to confirm the significance of the periodicity and rhythmicity of the miRNAs validated by RT-qPCR [27]. The miRNAs that exhibited *p*-values of less than 0.05 were considered rhythmic. All the statistical analyses were performed using R programming language.

### 4.7. Quantitative RT-qPCR for miRNA Expression

Reverse transcription of cyRNA time samples (performed separately in triplicate) was conducted using miRNA-specific stem-loop RT primers (see Table 3), which featured a 3′ overhang of 6 or 7 nucleotides complementary to the 3′ portion of the associated mature miRNA sequence [55]. We made a pool of seven stem-loop RT primers that included one endogenous control at 10 nM, allowing simultaneous reverse transcriptions in one tube [56]. Reverse transcriptase reactions contained 200 ng of cyRNA, a stem-loop RT primer pool, and reagents from a TaqMan MicroRNA Reverse Transcription Kit (Thermo Fisher Scientific) according to the manufacturer’s instructions. A negative control (no template) was included in all reactions.

Next, we performed pre-amplification of cDNA prior to quantification [57,58]. We facilitated two separate pre-amplification reactions using two miRNA primer pools. The first primer pool contained miR-1225-5p, miR-548ay-3p, and miR-769-3p, and the second contained miR-141-5p, miR-222-5p, and miR-17-5p. Both pools included miR-106a-5p as an endogenous control. For each reaction, 3 μL of cDNA, 100 nM of the miRNA primer pool (forward primers), 12.5 µL of 2× Universal Master Mix with no UNG (Thermo Fisher Scientific), 1 uM of universal reverse primer, 1 µL of 5 U/µL AmpliTaq Gold (Promega Corp, Madison, WI, USA), 0.5 µL of 100 mM dNTPs (Promega Corp), 0.5 µL of 100 mM MgCl2 (Promega Corp), and molecular grade water were used to achieve a final volume of 25 µL. Regarding the temperature profile of the reaction, incubation at 95 °C for 10 m, was followed by incubation at 55 °C for 2 m and 14 cycles of 95 °C for 1 s and 65 °C for 1 m. A negative pre-amplified control (no-cDNA) was included. Next, both pre-amplified cDNAs were diluted 10 times using molecular grade water and stored at −20 °C before further processing.

Finally, the qPCR was run using an Applied Biosystems^®^ StepOne™ Real-Time PCR System (Thermo Fisher Scientific). Seven miRNAs were profiled using 1X Brilliant II Fast SYBR^®^ Green qPCR Master Mix (Agilent Technologies). The reaction consisted of 1 µL of pre-amplified cDNA, 150 nM of forward and reverse primers (Table 3), 300 nM of passive reference dye, and molecular grade water to achieve a final volume of 12 µL. The following three-step cycling program was used: 95 °C for 3 min and 40 cycles of 95 °C for 6 s, 55 °C for 12 s, and 70 °C for 10 s. Melting curves were performed to ensure an efficient reaction. The relative expression was calculated using the ΔΔ*C*_t_ [48], with miR-106a-5p used for normalization [15]. The level of expression of each biological replicate was calculated separately and averaged, and the SEM was calculated for each time point. The relative expression results of each miRNA were plotted over 48 h at 4 h intervals.

### 4.8. Identification of Targeted mRNAs and Pathway Analysis

The MultiMiR package (version 1.0.1; http://multimir.ucdenver.edu/) was established to identify targeted miRNAs that were experimentally validated [28]. The Reactome pathway analysis tool (www.reactome.org/PathwayBrowser/#TOOL=AT) was used to perform annotation enrichment analysis using lists of targeted mRNAs [59].

## 5. Conclusions

Our results indicate that serum shock entrainment of breast cell lines induces rhythmic fluctuations in some miRNAs. Additionally, some miRNAs exhibit different expression profiles across breast cell lines, which suggests that tumorigenic and non-tumorigenic cells respond differently to serum shock in terms of transcription. Our results indirectly link the hypothesis that miRNAs may play a potential role in circadian architecture of breast cells. Further studies are required to understand the possible effects of the rhythmicity of miRNAs in breast cells and determine whether they can be used as potential biomarkers.

## Figures and Tables

**Table 1:** Rhythmic features of the miRNA expression profiles obtained over 48 h from the MetaCycle algorithm for non-tumorigenic and tumorigenic breast cell lines.

miRNA	*p*-Value	Period (h)	Phase (h)	Amplitude
MCF-10A				
hsa-miR-141-5p	0.0239 *	20.72	19.97	2.3
hsa-miR-1225-5p	0.9901	26.91	9.12	0.71
hsa-miR-17-5p	0.9659	27.06	4.73	3.79
hsa-miR-769-3p	0.9439	25.33	6.79	1.95
hsa-miR-222-5p	0.0174 *	20.02	0.40	3.91
hsa-miR-548ay-3p	0.0219 *	21.33	20.56	3.2
MCF-7				
hsa-miR-141-5p	0.0012 *	20.34	17.88	1.06
hsa-miR-1225-5p	0.9526	28.67	1.53	0.89
hsa-miR-17-5p	0.9050	25.04	19.81	2.24
hsa-miR-769-3p	0.0205 *	20.19	2.26	1.2
hsa-miR-222-5p	0.0492 *	22.75	13.95	0.94
hsa-miR-548ay-3p	0.6690	23.87	1.99	1.63
MDA-MB-231				
hsa-miR-141-5p	0.9372	22.46	13.76	0.99
hsa-miR-1225-5p	0.6841	25.33	4.72	0.99
hsa-miR-17-5p	0.0018 *	24.90	20.16	3.53
hsa-miR-769-3p	0.0214 *	22.09	19.90	2.55
hsa-miR-222-5p	0.7651	25.80	7.37	1.06
hsa-miR-548ay-3p	0.0027 *	22.73	1.09	1
ZR-7530				
hsa-miR-141-5p	0.2357	24.52	15.42	3.68
hsa-miR-1225-5p	0.0079 *	24.69	13.19	1.6
hsa-miR-17-5p	0.6695	25.47	16.15	2.69
hsa-miR-769-3p	0.9661	28.67	14.22	1.76
hsa-miR-222-5p	0.1897	27.15	7.89	2.24
hsa-miR-548ay-3p	0.00001 *	24.56	12.47	2.63
HCC-1954				
hsa-miR-141-5p	0.0411 *	23.28	9.51	4.35
hsa-miR-1225-5p	0.0373 *	23.99	21.86	1
hsa-miR-17-5p	0.0380 *	22.32	8.14	7.87
hsa-miR-769-3p	0.9610	21.33	18.46	4.65
hsa-miR-222-5p	0.9907	28.67	7.65	6.22
hsa-miR-548ay-3p	0.0040 *	23.64	21.33	1.36

* *p* < 0.05 around 24 h.

**Table 2:** List of primers for clock genes.

Gene	Primer	Sequence (5′→3′)	NCBI RefSeq
*BMAL1*	Forward	CATTGTGCACAGAAGCATCA	NM_001178.4
	Reverse	ACAAGGAAGAATAAACGGCTTT	
*PER2*	Forward	TGCCAAAATCTTACTCTGCTG	NM_022817.2
	Reverse	GGCATCACGTAAACAAATTCA	
*SERPINB1*	Forward	AGGTTCATTCAAGATTCCAGAGT	NM_030666.3 70
	Reverse	AGTTTCAGAATATAAGACGCTCCA	
*GAPDH*	Forward	AGCCACATCGCTCAGACAC	NM_002046.4
	Reverse	TGGCAACAATATCCACTTTACCAGA	

**Table 3:** List of primers for miRNA validation.

miRNA	Primer	Sequence (5′→3′)	Mature Accession Number
hsa-miR-141-5p	Stem-loop	GTC GTA TCC AGT GCA GGG TCC GAG GTA TTC GCA CTG GAT ACG AC TCCAAC	MIMAT0004598
	Forward	CAC GCA CAT CTT CCA GTA C	
hsa-miR-1225-5p	Stem-loop	GTC GTA TCC AGT GCA GGG TCC GAG GTA TTC GCA CTG GAT ACG AC CCCCCC	MIMAT0005572
	Forward	CAACAGTGGGTACGGCCCA	
hsa-miR-17-5p	Stem-loop	GTC GTA TCC AGT GCA GGG TCC GAG GTA TTC GCA CTG GAT ACG AC CTACCT	MIMAT0000070
	Forward	CAC GCA CAA AGT GCT TAC A	
hsa-miR-769-3p	Stem-loop	GTC GTA TCC AGT GCA GGG TCC GAG GTA TTC GCA CTG GAT ACG AC AACCAA	MIMAT0003887
	Forward	CAA CAC TGG GAT CTC CGG	
hsa-miR-222-5p	Stem-loop	GTC GTA TCC AGT GCA GGG TCC GAG GTA TTC GCA CTG GAT ACG AC AGGATC	MIMAT0004569
	Forward	CAG CAC TCA GTA GCC AGT	
hsa-miR-548ay-3p	Stem-loop	GTC GTA TCC AGT GCA GGG TCC GAG GTA TTC GCA CTG GAT ACG AC TGCAAG	MIMAT0025453
	Forward	CAG CAC AAA ACC GCG AT	
hsa-miR-106a-5p	Stem-loop	GTC GTA TCC AGT GCA GGG TCC GAG GTA TTC GCA CTG GAT ACG AC GCTACC	MIMAT0000103
	Forward	CAC GCA AAAAGTGCTTACAGT	
	Universal	TCG TA TCC AGT GCA GGG T	

## Supplementary Materials

Supplementary materials can be found at www.mdpi.com/1422-0067/18/7/1499/s1.

## Abbreviations

BMAL1	also known arntl, aryl hydrocarbon receptor nuclear translocator like
PER2	Period circadian clock 2
SERPINB1	Serpin family B member 1
TL	Time-label
RW	Row-wise
CW	Column-wise
RCW	Row-column-wise
RCWB	Row-column-wise by blocks
