# Supplementary material for: Entrainment of Breast Cell Lines Results in Rhythmic Fluctuations of MicroRNAs
Source: Int J Mol Sci. 2017 Jul 12;18(7):1499. doi: 10.3390/ijms18071499 (PMC5535989; doi:10.3390/ijms18071499)
Supplement: Supplementary file 1 [file ijms-18-01499-s001.pdf]

Supplementary Material: Rhythmic expression patterns of microRNAs in human breast cell lines

Rafael Chacolla-Huaringa, Jorge Moreno-Cuevas, Victor Trevino-Alvarado and Sean-Patrick Scott

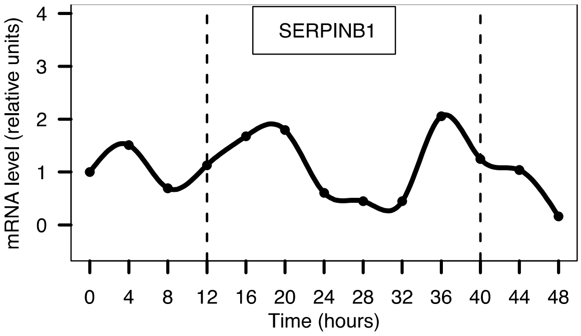

Supplementary Figure S1. Expression profiles of SERPINB1 gene in MCF-7. The profile expression of SERPINB1 gene was done by RT-qPCR in serum-shocked MCF-7 cells during 48 hours (4-hour intervals). Data points (mean of triplicates ± SEM) were normalized using GAPDH gene relative to the first time point (t = 0). Dotted gray lines at 12-h and 40h were added to show the period where profiles have robustness.

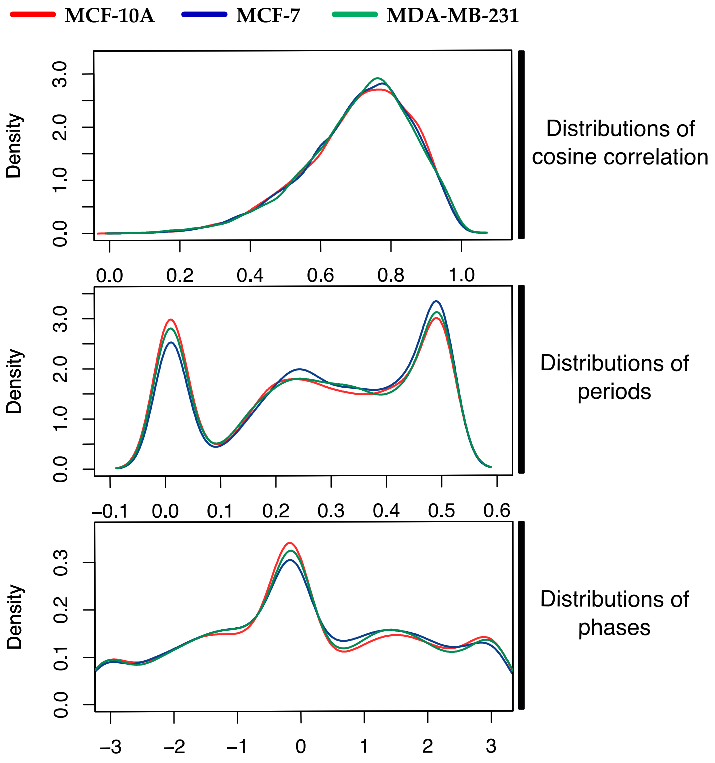

Supplementary Figure S2 Cosine correlation, period and phase distributions for MCF-10[a], MCF-7 and MDA-MB-231 microarray data. Panel (A) shows the distributions of cosine correlation, panel (B) period, and panel (C) phase obtained from microarray data of three tested breast cell lines.

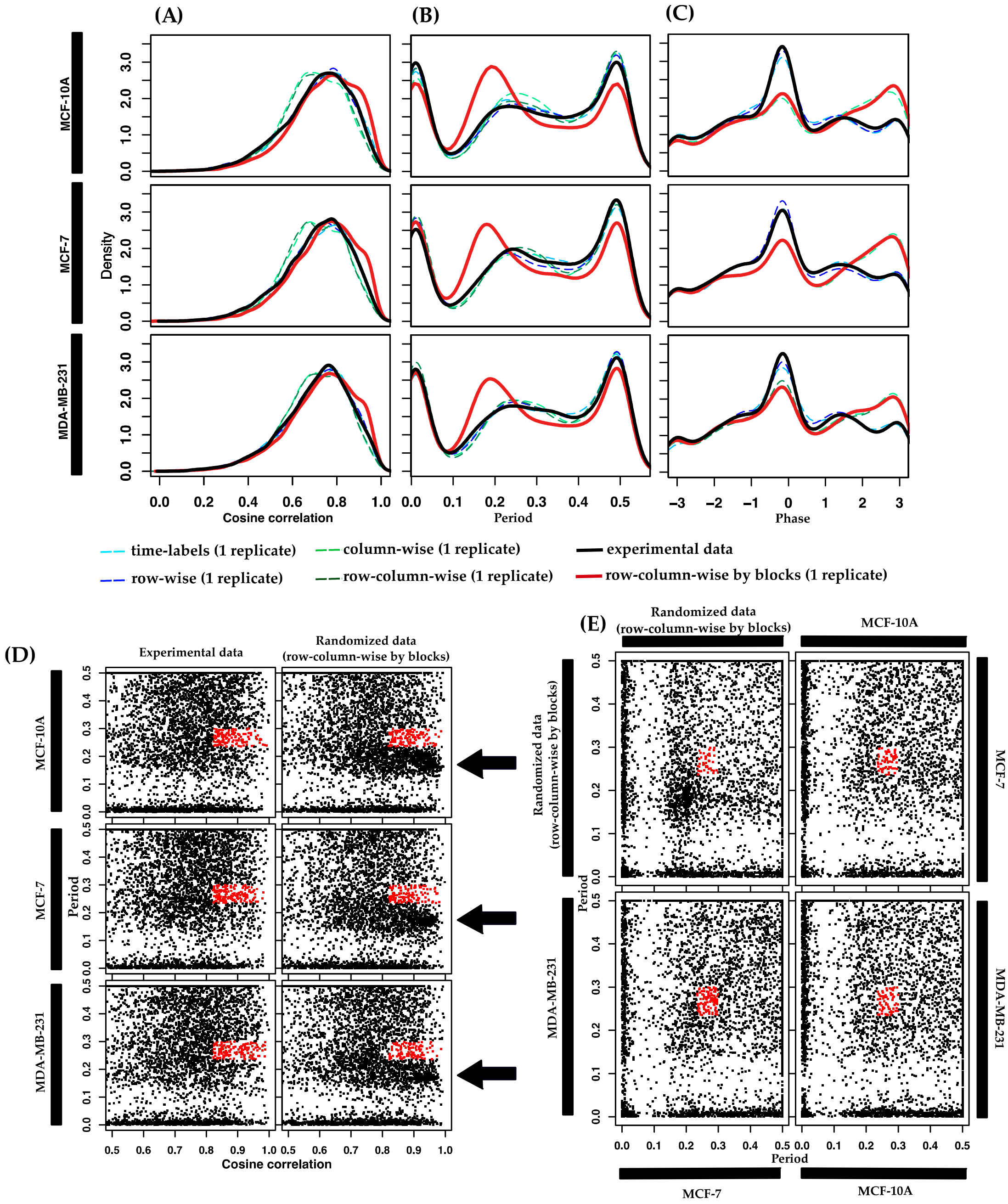

Supplementary Figure S3. Evaluation of the cosine correlation, period and phase distributions in experimental and randomized data. Experimental data consisted of the microarrays data for MCF-10A, MCF-7 and MDA-MB-231. Randomized data consisted of five types of data (by triplicate) generated from 5 types of randomization methods: TL, CW, RW, CRW and CRWB. Panel (A) shows the distributions of cosine correlation, panel (B) period, and panel (C) phase among experimental and randomized data for the three tested breast cell lines. Panel (D) shows cosine correlation compared to the period from experimental and randomized (CRWB) data. An arrow mark regions in which miRNAs with large cosine correlation is highly associated to non-periodic values in randomized experiments whereas experimental data do not show such associations. Panel (E) shows plots of the period values in experimental and randomized (CRWB) data (two replicates). Red dots represent miRNAs fulfilling the selection criteria in correlation and period values.

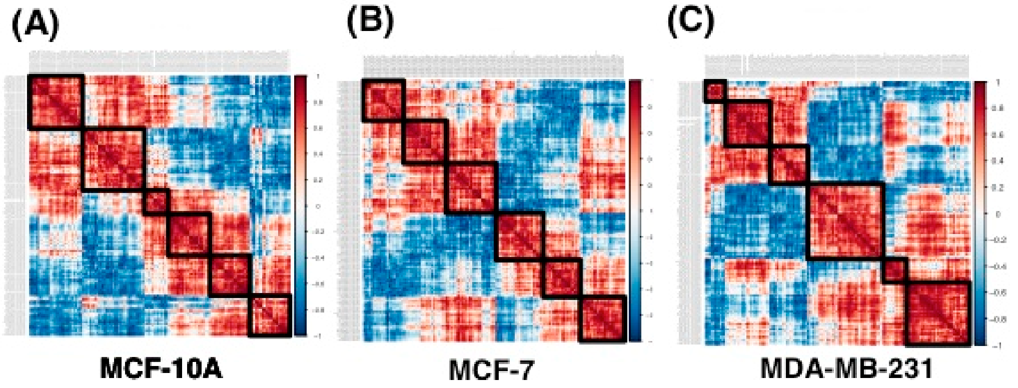

Supplementary Figure S4. Correlation plots of MCF-10A, MCF-7 and MDA-MB-231 cells. They illustrate the disposition of the clusters identified for each breast cell line.

Table S1. Circadian characteristics of the miRNA expression profiles obtained from cosine-fitting function in a period of 28 hours for MCF-10A.

miRNA	probe ID	cosine correlation	period	phase	amplitude	cluster
miR-1273e	A_25_P00016226	0.837	0.241	1.122	0.16	1
miR-191-3p	A_25_P00010878	0.883	0.237	1.104	0.19	1
miR-196b-3p	A_25_P00015383	0.867	0.283	1.475	0.07	1
miR-204-3p	A_25_P00017403	0.830	0.289	0.753	0.06	1
miR-20b-3p	A_25_P00013574	0.842	0.248	0.891	0.16	1
miR-23c	A_25_P00016198	0.875	0.276	1.928	0.06	1
miR-27b-5p	A_25_P00013377	0.846	0.235	0.988	0.16	1
miR-323b-5p	A_25_P00012440	0.849	0.286	1.195	0.11	1
miR-3622b-3p	A_25_P00016091	0.895	0.257	0.658	0.06	1
miR-3687	A_25_P00016033	0.823	0.237	1.654	0.08	1
miR-3689f	A_25_P00017126	0.821	0.233	2.284	0.06	1
miR-3908	A_25_P00015990	0.829	0.274	0.341	0.08	1
miR-4253	A_25_P00015539	0.830	0.269	0.149	0.08	1
miR-4456	A_25_P00016281	0.894	0.245	1.128	0.09	1
miR-4652-3p	A_25_P00017178	0.841	0.254	1.396	0.13	1
miR-4738-5p	A_25_P00016564	0.824	0.255	1.077	0.11	1
miR-548aa	A_25_P00016262	0.850	0.270	1.065	0.07	1
miR-548am-5p	A_25_P00012797	0.894	0.272	0.537	0.09	1
miR-548b-3p	A_25_P00010122	0.828	0.240	1.190	0.08	1
miR-552-3p	A_25_P00011479	0.851	0.240	1.323	0.11	1
miR-554	A_25_P00011289	0.916	0.249	0.834	0.08	1
miR-5572	A_25_P00017423	0.847	0.280	1.529	0.08	1
miR-5581-3p	A_25_P00017621	0.892	0.227	1.201	0.05	1
miR-760	A_25_P00013023	0.934	0.300	0.005	0.05	1
miR-10a-3p	A_25_P00013300	0.872	0.278	-0.176	0.08	2
miR-133a-3p	A_25_P00012167	0.830	0.239	0.496	0.06	2

miR-216b-5p	A_25_P00013029	0.835	0.232	0.740	0.08	2
miR-3147	A_25_P00015806	0.964	0.275	-0.068	0.07	2
miR-3169	A_25_P00015687	0.958	0.269	0.158	0.1	2
miR-34c-3p	A_25_P00012289	0.902	0.257	0.414	0.11	2
miR-3687	A_25_P00016032	0.881	0.232	0.792	0.05	2
miR-374b-3p	A_25_P00013680	0.909	0.270	-0.343	0.12	2
miR-4309	A_25_P00015857	0.931	0.229	0.102	0.11	2
miR-432-3p	A_25_P00010355	0.914	0.252	0.122	0.06	2
miR-4529-5p	A_25_P00017271	0.973	0.225	0.892	0.14	2
miR-4724-3p	A_25_P00016410	0.837	0.235	0.786	0.05	2
miR-4795-3p	A_25_P00016548	0.917	0.251	0.441	0.1	2
miR-503-5p	A_25_P00010658	0.844	0.282	-0.268	0.08	2
miR-519e-3p	A_25_P00012524	0.995	0.226	0.666	0.11	2
miR-5580-3p	A_25_P00017645	0.971	0.262	0.027	0.07	2
miR-5692b	A_25_P00017386	0.911	0.257	0.272	0.11	2
miR-6082	A_25_P00017900	0.852	0.252	0.302	0.05	2
miR-647	A_25_P00011370	0.930	0.296	-0.447	0.2	2
miR-873-5p	A_25_P00013008	0.837	0.289	0.008	0.19	2
miR-1271-5p	A_25_P00015043	0.928	0.242	-0.252	0.09	3
miR-150-5p	A_25_P00014847	0.884	0.299	-1.240	0.16	3
miR-20a-3p	A_25_P00013170	0.942	0.276	-0.940	0.06	3
miR-297	A_25_P00013108	0.970	0.258	-0.566	0.09	3
miR-3137	A_25_P00015538	0.969	0.248	-0.737	0.06	3
miR-3166	A_25_P00015802	0.882	0.290	-0.860	0.07	3
miR-3178	A_25_P00015670	0.845	0.229	0.148	0.09	3
miR-3617-5p	A_25_P00016108	0.860	0.274	-0.513	0.11	3
miR-383-5p	A_25_P00010384	0.838	0.280	-0.671	0.07	3
miR-4633-3p	A_25_P00017193	0.824	0.299	-1.091	0.06	3
miR-4652-5p	A_25_P00017075	0.854	0.259	-0.085	0.05	3
miR-4673	A_25_P00017120	0.906	0.278	-0.664	0.08	3
miR-4695-5p	A_25_P00016742	0.832	0.287	-0.659	0.08	3
miR-509-3p	A_25_P00012678	0.852	0.255	0.062	0.05	3
miR-548a-3p	A_25_P00014240	0.894	0.294	-1.183	0.18	3
miR-5683	A_25_P00017565	0.980	0.243	-0.068	0.08	3
miR-6072	A_25_P00017833	0.839	0.274	-0.872	0.05	3
miR-615-3p	A_25_P00012788	0.839	0.258	-0.444	0.16	3
miR-657	A_25_P00011359	0.842	0.296	-1.090	0.24	3
miR-888-3p	A_25_P00013661	0.885	0.296	-1.024	0.06	3
miR-888-5p	A_25_P00012939	0.892	0.268	-0.207	0.12	3
miR-9-5p	A_25_P00011003	0.846	0.253	-1.001	0.06	3
miR-942-5p	A_25_P00013097	0.919	0.280	-0.897	0.08	3
dmr_285		0.842	0.280	-1.603	0.06	4
miR-2277-5p	A_25_P00016164	0.833	0.293	-1.733	0.07	4

miR-27a-5p	A_25_P00013203	0.986	0.241	-0.945	0.16	4
miR-3607-3p	A_25_P00016097	0.822	0.275	-1.539	0.06	4
miR-3935	A_25_P00016203	0.881	0.239	-1.200	0.09	4
miR-450b-5p	A_25_P00012908	0.924	0.280	-1.607	0.07	4
miR-4746-3p	A_25_P00017145	0.872	0.295	-1.246	0.08	4
miR-548m	A_25_P00015184	0.847	0.241	-0.873	0.08	4
miR-589-5p	A_25_P00012761	0.860	0.272	-1.203	0.06	4
miR-6511a-5p	A_25_P00017819	0.956	0.291	-1.880	0.08	4
miR-6715b-5p	A_25_P00017971	0.853	0.254	-1.332	0.08	4
miR-889-3p	A_25_P00012949	0.835	0.239	-0.937	0.17	4
miR-944	A_25_P00013105	0.927	0.233	-1.170	0.16	4
let-7g-3p	A_25_P00013362	0.974	0.261	-2.109	0.07	5
miR-1269b	A_25_P00016688	0.821	0.261	-1.987	0.08	5
miR-140-3p	A_25_P00012176	0.914	0.260	-2.683	0.05	5
miR-141-5p	A_25_P00013414	0.826	0.266	-2.300	0.23	5
miR-193a-3p	A_25_P00012258	0.952	0.234	-1.737	0.12	5
miR-222-5p	A_25_P00013351	0.916	0.258	-2.284	0.08	5
miR-224-3p	A_25_P00015401	0.837	0.281	-2.386	0.09	5
miR-301a-5p	A_25_P00017444	0.830	0.252	-2.386	0.08	5
miR-302b-3p	A_25_P00010618	0.853	0.267	-2.071	0.16	5
miR-3129-3p	A_25_P00016905	0.829	0.263	-1.639	0.12	5
miR-3152-3p	A_25_P00015884	0.897	0.247	-1.536	0.05	5
miR-330-5p	A_25_P00012346	0.857	0.231	-1.614	0.05	5
miR-3615	A_25_P00016166	0.926	0.226	-1.627	0.06	5
miR-363-3p	A_25_P00010953	0.860	0.230	-1.793	0.06	5
miR-3681-5p	A_25_P00016174	0.853	0.257	-1.663	0.06	5
miR-3942-3p	A_25_P00016582	0.855	0.270	-2.587	0.11	5
miR-3973	A_25_P00016792	0.864	0.234	-1.318	0.06	5
miR-4286	A_25_P00015773	0.923	0.251	-2.734	0.27	5
miR-4511	A_25_P00016505	0.837	0.271	-2.782	0.07	5
miR-4697-5p	A_25_P00016630	0.904	0.279	-2.331	0.05	5
miR-4760-5p	A_25_P00016788	0.884	0.251	-2.418	0.13	5
miR-4766-5p	A_25_P00016303	0.840	0.291	-2.645	0.07	5
miR-4804-3p	A_25_P00017321	0.836	0.289	-2.540	0.07	5
miR-515-5p	A_25_P00010499	0.835	0.251	-2.071	0.12	5
miR-548ay-3p	A_25_P00017966	0.942	0.284	-2.360	0.05	5
miR-5572	A_25_P00017424	0.992	0.238	-1.505	0.05	5
miR-766-3p	A_25_P00011410	0.863	0.236	-1.501	0.11	5
miR-891a-5p	A_25_P00012881	0.831	0.266	-1.823	0.12	5
miR-921	A_25_P00013042	0.880	0.225	-1.570	0.08	5
miR-127-5p	A_25_P00012219	0.929	0.252	-2.529	0.07	6
miR-129-5p	A_25_P00013880	0.951	0.238	-2.271	0.06	6
miR-132-5p	A_25_P00013400	0.879	0.232	-1.832	0.05	6

miR-181b-5p	A_25_P00012089	0.821	0.225	-2.917	0.07	6
miR-200a-5p	A_25_P00011010	0.859	0.250	-2.114	0.07	6
miR-212-3p	A_25_P00010854	0.827	0.226	-2.944	0.12	6
miR-29a-5p	A_25_P00013209	0.871	0.285	3.142	0.05	6
miR-3177-5p	A_25_P00016485	0.860	0.245	-3.142	0.06	6
miR-3202	A_25_P00015596	0.888	0.278	-2.608	0.06	6
miR-324-5p	A_25_P00010153	0.863	0.264	-2.810	0.05	6
miR-34a-3p	A_25_P00013311	0.919	0.239	-2.585	0.09	6
miR-3612	A_25_P00016230	0.849	0.224	-3.142	0.12	6
miR-3619-5p	A_25_P00016183	0.957	0.235	-2.010	0.14	6
miR-367-3p	A_25_P00010985	0.902	0.238	-2.295	0.08	6
miR-3674	A_25_P00016190	0.872	0.260	-2.534	0.06	6
miR-371a-3p	A_25_P00013992	0.864	0.279	3.138	0.07	6
miR-3939	A_25_P00016163	0.821	0.270	2.929	0.05	6
miR-3978	A_25_P00016369	0.847	0.295	-2.352	0.09	6
miR-4254	A_25_P00015728	0.881	0.280	2.619	0.12	6
miR-4432	A_25_P00016498	0.885	0.289	-2.494	0.06	6
miR-4440	A_25_P00016616	0.832	0.290	2.781	0.06	6
miR-4498	A_25_P00016649	0.842	0.242	-2.719	0.05	6
miR-4652-3p	A_25_P00017179	0.866	0.248	-2.467	0.09	6
miR-4723-5p	A_25_P00017205	0.887	0.258	-2.245	0.05	6
miR-4803	A_25_P00017101	0.854	0.277	3.089	0.08	6
miR-5006-3p	A_25_P00017548	0.929	0.236	-3.142	0.07	6
miR-501-3p	A_25_P00012640	0.853	0.295	-3.075	0.09	6
miR-548au-5p	A_25_P00017584	0.836	0.254	-3.032	0.07	6
miR-548b-5p	A_25_P00012756	0.933	0.269	-2.969	0.11	6
miR-5588-3p	A_25_P00017615	0.852	0.256	-2.407	0.06	6
miR-646	A_25_P00011963	0.845	0.245	-2.894	0.18	6
miR-891a-5p	A_25_P00012882	0.936	0.262	-2.305	0.07	6
miR-943	A_25_P00013101	0.945	0.238	-1.906	0.06	6
miR-98-3p	A_25_P00017910	0.849	0.274	2.601	0.05	6

Table S2. Circadian characteristics of the miRNA expression profiles obtained from cosine-fitting function in a period of 28 hours for MCF-7.

miRNA	agilent probe ID	cosine correlation	period	phase	amplitude	cluster
miR-10a-3p	A_25_P00013302	0.828	0.241	1.026	0.13	1
miR-1185-1-3p	A_25_P00017447	0.943	0.237	2.010	0.08	1
miR-1207-5p	A_25_P00015088	0.825	0.224	2.131	0.11	1
miR-1225-5p	A_25_P00014920	0.949	0.225	1.879	0.23	1
miR-1234-5p	A_25_P00017828	0.842	0.281	1.922	0.4	1
miR-1299	A_25_P00015122	0.863	0.275	0.921	0.08	1
miR-28-3p	A_25_P00012006	0.846	0.258	0.947	0.12	1
miR-3121-3p	A_25_P00015873	0.837	0.248	1.300	0.06	1
miR-3165	A_25_P00015847	0.888	0.265	1.253	0.07	1
miR-320e	A_25_P00015664	0.859	0.293	0.493	0.08	1
miR-3620-3p	A_25_P00016148	0.863	0.226	1.737	0.07	1
miR-378j	A_25_P00017982	0.847	0.281	1.183	0.09	1
miR-3928-3p	A_25_P00016175	0.880	0.253	2.115	0.05	1
miR-4294	A_25_P00015651	0.902	0.268	1.844	0.05	1
miR-4472	A_25_P00016275	0.877	0.253	1.407	0.09	1
miR-4507	A_25_P00016700	0.911	0.248	1.795	0.28	1
miR-4530	A_25_P00016774	0.921	0.224	2.208	0.38	1
miR-4534	A_25_P00016446	0.836	0.290	1.840	0.18	1
miR-4639-3p	A_25_P00016894	0.882	0.234	1.641	0.05	1
miR-4672	A_25_P00017202	0.831	0.237	1.962	0.44	1
miR-4699-3p	A_25_P00016970	0.826	0.261	2.418	0.09	1
miR-4701-3p	A_25_P00016366	0.922	0.243	1.161	0.05	1
miR-4712-3p	A_25_P00016524	0.952	0.281	1.734	0.09	1
miR-4725-5p	A_25_P00017000	0.894	0.297	0.678	0.05	1
miR-4764-3p	A_25_P00016695	0.861	0.243	1.425	0.06	1
miR-4787-5p	A_25_P00016992	0.831	0.274	1.320	0.07	1
miR-4793-5p	A_25_P00016728	0.864	0.239	0.989	0.06	1
miR-548b-5p	A_25_P00012756	0.840	0.266	1.278	0.12	1
miR-549a	A_25_P00010365	0.901	0.249	1.333	0.12	1
miR-562	A_25_P00011389	0.897	0.269	0.737	0.16	1
miR-6087	A_25_P00017892	0.837	0.255	2.388	0.49	1
miR-764	A_25_P00015440	0.834	0.262	1.129	0.07	1
miR-940	A_25_P00013090	0.833	0.299	0.606	0.05	1
miR-10a-3p	A_25_P00013301	0.898	0.253	0.326	0.08	2
miR-1206	A_25_P00015086	0.827	0.276	0.096	0.09	2
miR-1238-5p	A_25_P00017772	0.838	0.279	0.128	0.06	2
miR-127-5p	A_25_P00012221	0.865	0.299	-0.246	0.1	2
miR-1304-3p	A_25_P00017635	0.839	0.235	0.832	0.08	2
miR-188-5p	A_25_P00012246	0.852	0.294	0.406	0.07	2

miR-2116-3p	A_25_P00015463	0.832	0.261	0.565	0.11	2
miR-222-3p	A_25_P00012126	0.854	0.294	-0.450	0.19	2
miR-3185	A_25_P00015654	0.918	0.263	0.336	0.1	2
miR-335-3p	A_25_P00013557	0.948	0.275	0.138	0.05	2
miR-3944-5p	A_25_P00017159	0.829	0.254	0.206	0.07	2
miR-4313	A_25_P00015772	0.837	0.261	0.375	0.06	2
miR-452-3p	A_25_P00013583	0.890	0.235	0.323	0.07	2
miR-4520-2-3p	A_25_P00016398	0.890	0.287	-0.112	0.05	2
miR-4529-5p	A_25_P00017271	0.864	0.269	0.458	0.05	2
miR-4723-5p	A_25_P00017205	0.867	0.233	0.535	0.08	2
miR-5047	A_25_P00016399	0.845	0.253	0.567	0.1	2
miR-525-3p	A_25_P00012549	0.896	0.234	0.684	0.07	2
miR-548x-3p	A_25_P00016860	0.964	0.240	0.613	0.06	2
miR-550a-5p	A_25_P00012768	0.909	0.240	0.810	0.18	2
miR-554	A_25_P00011288	0.853	0.250	0.665	0.18	2
miR-5704	A_25_P00017598	0.870	0.256	0.662	0.08	2
miR-708-3p	A_25_P00013671	0.865	0.297	-0.090	0.09	2
miR-744-3p	A_25_P00013675	0.879	0.260	0.519	0.24	2
miR-876-5p	A_25_P00012961	0.831	0.234	1.041	0.12	2
miR-935	A_25_P00013071	0.825	0.285	-0.272	0.09	2
miR-1262	A_25_P00015178	0.950	0.233	-0.215	0.05	3
miR-129-1-3p	A_25_P00013277	0.893	0.262	-0.846	0.06	3
miR-1295b-3p	A_25_P00017728	0.891	0.300	-1.082	0.06	3
miR-181d-5p	A_25_P00012514	0.823	0.289	-0.820	0.11	3
miR-191-5p	A_25_P00012203	0.837	0.296	-1.556	0.08	3
miR-203b-3p	A_25_P00017122	0.939	0.243	-0.070	0.05	3
miR-219b-5p	A_25_P00016753	0.935	0.293	-0.609	0.08	3
miR-222-5p	A_25_P00013351	0.979	0.252	-0.427	0.07	3
miR-3123	A_25_P00015736	0.871	0.276	-1.069	0.05	3
miR-3182	A_25_P00015725	0.888	0.268	-0.476	0.09	3
miR-32-5p	A_25_P00012021	0.899	0.257	-1.006	0.12	3
miR-3622a-3p	A_25_P00016014	0.854	0.245	-0.612	0.08	3
miR-367-3p	A_25_P00010984	0.867	0.284	-1.240	0.07	3
miR-412-3p	A_25_P00010266	0.869	0.252	-0.311	0.16	3
miR-4427	A_25_P00016416	0.938	0.281	-1.257	0.08	3
miR-4429	A_25_P00016765	0.923	0.283	-1.753	0.07	3
miR-4433-5p	A_25_P00017390	0.866	0.273	-0.553	0.05	3
miR-4441	A_25_P00017055	0.987	0.234	-0.543	0.05	3
miR-4643	A_25_P00017260	0.827	0.274	-0.635	0.08	3
miR-4743-5p	A_25_P00016575	0.884	0.260	-0.490	0.08	3
miR-4764-5p	A_25_P00016450	0.821	0.282	-1.015	0.11	3
miR-4769-3p	A_25_P00017011	0.836	0.273	-1.318	0.1	3
miR-4786-5p	A_25_P00016997	0.924	0.300	-1.906	0.05	3

miR-499b-5p	A_25_P00017168	0.862	0.290	-0.732	0.08	3
miR-542-5p	A_25_P00012858	0.879	0.293	-0.878	0.07	3
miR-548az-5p	A_25_P00017868	0.951	0.280	-0.900	0.08	3
miR-551a	A_25_P00011473	0.941	0.239	-0.694	0.07	3
miR-551a	A_25_P00011638	0.953	0.254	-0.527	0.09	3
miR-553	A_25_P00011219	0.825	0.294	-1.794	0.12	3
miR-556-3p	A_25_P00012717	0.919	0.266	-0.864	0.08	3
miR-614	A_25_P00010535	0.867	0.223	-0.231	0.09	3
miR-628-5p	A_25_P00012814	0.838	0.297	-1.093	0.07	3
miR-6505-3p	A_25_P00017858	0.930	0.256	-0.853	0.1	3
miR-106b-3p	A_25_P00013479	0.846	0.281	-1.044	0.13	4
miR-1184	A_25_P00015066	0.910	0.276	-1.799	0.06	4
miR-1225-3p	A_25_P00014924	0.884	0.245	-0.967	0.1	4
miR-1236-3p	A_25_P00014955	0.955	0.267	-1.833	0.09	4
miR-1236-3p	A_25_P00014956	0.879	0.261	-1.480	0.09	4
miR-155-3p	A_25_P00013471	0.899	0.262	-1.900	0.07	4
miR-16-1-3p	A_25_P00013145	0.890	0.270	-1.836	0.07	4
miR-191-5p	A_25_P00012202	0.883	0.269	-1.784	0.17	4
miR-223-5p	A_25_P00013355	0.834	0.224	-0.963	0.08	4
miR-302d-5p	A_25_P00013516	0.853	0.279	-1.706	0.13	4
miR-30b-3p	A_25_P00013381	0.832	0.280	-1.961	0.06	4
miR-30c-2-3p	A_25_P00013287	0.919	0.297	-2.394	0.05	4
miR-32-3p	A_25_P00013217	0.958	0.241	-1.072	0.08	4
miR-363-5p	A_25_P00010043	0.922	0.263	-1.348	0.09	4
miR-3908	A_25_P00015990	0.877	0.253	-0.824	0.08	4
miR-4419a	A_25_P00017190	0.830	0.231	-1.450	0.07	4
miR-4537	A_25_P00016657	0.908	0.253	-1.535	0.14	4
miR-4663	A_25_P00016419	0.878	0.256	-1.388	0.06	4
miR-5089-5p	A_25_P00017582	0.827	0.228	-1.517	0.1	4
miR-518c-5p	A_25_P00012559	0.948	0.245	-0.972	0.06	4
miR-548ae-3p	A_25_P00017682	0.879	0.280	-1.601	0.11	4
miR-548at-5p	A_25_P00017425	0.929	0.266	-1.833	0.09	4
miR-5584-3p	A_25_P00017368	0.955	0.257	-1.430	0.05	4
miR-610	A_25_P00011025	0.836	0.255	-1.451	0.13	4
miR-620	A_25_P00010450	0.854	0.236	-0.977	0.06	4
miR-769-3p	A_25_P00011231	0.962	0.223	-1.144	0.16	4
miR-892c-3p	A_25_P00017937	0.923	0.269	-1.593	0.11	4
miR-105-5p	A_25_P00012041	0.847	0.246	-1.730	0.09	5
miR-1273c	A_25_P00015723	0.830	0.223	-1.123	0.1	5
miR-138-1-3p	A_25_P00013440	0.892	0.240	-1.702	0.16	5
miR-152-3p	A_25_P00012196	0.880	0.249	-2.018	0.07	5
miR-187-5p	A_25_P00013327	0.929	0.235	-1.825	0.09	5
miR-193b-5p	A_25_P00013597	0.899	0.262	-2.359	0.08	5

miR-202-3p	A_25_P00014864	0.825	0.224	-1.848	0.05	5
miR-206	A_25_P00010528	0.928	0.236	-1.863	0.07	5
miR-27b-5p	A_25_P00013378	0.861	0.235	-1.484	0.21	5
miR-302b-3p	A_25_P00010618	0.925	0.249	-1.929	0.07	5
miR-3132	A_25_P00015821	0.822	0.238	-1.781	0.07	5
miR-320a	A_25_P00012262	0.926	0.254	-2.209	0.05	5
miR-3622b-5p	A_25_P00016219	0.837	0.235	-1.291	0.06	5
miR-3650	A_25_P00016185	0.860	0.281	-2.343	0.05	5
miR-3654	A_25_P00016268	0.864	0.227	-1.210	0.05	5
miR-3660	A_25_P00016217	0.824	0.235	-1.818	0.09	5
miR-3688-3p	A_25_P00016048	0.870	0.226	-1.298	0.1	5
miR-4262	A_25_P00015626	0.892	0.241	-1.559	0.05	5
miR-4304	A_25_P00015850	0.928	0.226	-1.853	0.1	5
miR-4447	A_25_P00016283	0.916	0.265	-1.676	0.06	5
miR-4483	A_25_P00016607	0.824	0.295	-2.363	0.09	5
miR-4499	A_25_P00017132	0.831	0.233	-1.811	0.06	5
miR-4724-5p	A_25_P00016749	0.854	0.256	-2.459	0.08	5
miR-4758-5p	A_25_P00016654	0.847	0.243	-2.269	0.06	5
miR-4774-5p	A_25_P00017266	0.874	0.227	-1.906	0.08	5
miR-4789-5p	A_25_P00016661	0.846	0.277	-2.067	0.14	5
miR-488-5p	A_25_P00014633	0.950	0.248	-2.090	0.05	5
miR-548ab	A_25_P00016460	0.894	0.241	-1.904	0.09	5
miR-548ae-3p	A_25_P00017681	0.842	0.252	-2.102	0.16	5
miR-551b-5p	A_25_P00013620	0.914	0.268	-2.335	0.05	5
miR-5588-5p	A_25_P00017336	0.902	0.270	-2.352	0.05	5
miR-569	A_25_P00010313	0.882	0.227	-1.268	0.09	5
miR-6499-5p	A_25_P00017751	0.861	0.266	-1.897	0.06	5
miR-660-5p	A_25_P00010459	0.855	0.293	-2.955	0.09	5
miR-664a-3p	A_25_P00015251	0.887	0.291	-2.336	0.06	5
miR-99a-5p	A_25_P00010471	0.913	0.273	-2.390	0.07	5
miR-1179	A_25_P00015056	0.899	0.263	-2.990	0.05	6
miR-1228-5p	A_25_P00015006	0.910	0.240	-2.358	0.07	6
miR-1269b	A_25_P00016688	0.982	0.273	-2.827	0.05	6
miR-1468-5p	A_25_P00015296	0.905	0.271	-3.142	0.05	6
miR-148a-5p	A_25_P00013281	0.942	0.273	-2.902	0.06	6
miR-3115	A_25_P00015534	0.831	0.244	-2.415	0.06	6
miR-3156-3p	A_25_P00016435	0.882	0.249	-2.723	0.05	6
miR-3615	A_25_P00016166	0.859	0.248	-3.065	0.07	6
miR-363-5p	A_25_P00010041	0.838	0.263	-3.003	0.08	6
miR-4804-3p	A_25_P00017320	0.860	0.300	2.979	0.1	6
miR-483-5p	A_25_P00012459	0.858	0.277	2.279	0.05	6
miR-5190	A_25_P00017704	0.926	0.224	-2.994	0.06	6
miR-5197-3p	A_25_P00017465	0.926	0.232	-2.150	0.05	6

miR-526b-3p	A_25_P00010784	0.835	0.237	-2.798	0.08	6
miR-548at-5p	A_25_P00017427	0.890	0.247	-2.477	0.09	6
miR-548ay-3p	A_25_P00017966	0.916	0.234	-2.554	0.07	6
miR-548h-3p	A_25_P00016243	0.841	0.268	3.142	0.05	6
miR-548i	A_25_P00015225	0.937	0.277	-2.788	0.07	6
miR-5586-5p	A_25_P00017601	0.840	0.299	2.702	0.08	6
miR-570-3p	A_25_P00012721	0.891	0.225	-2.360	0.07	6
miR-580-3p	A_25_P00011353	0.877	0.297	2.246	0.08	6
miR-581	A_25_P00011283	0.839	0.256	-2.931	0.06	6
miR-6083	A_25_P00017802	0.926	0.292	2.390	0.07	6
miR-6128	A_25_P00017984	0.896	0.261	-2.655	0.09	6
miR-617	A_25_P00010772	0.836	0.294	3.142	0.1	6
miR-626	A_25_P00011235	0.945	0.291	2.518	0.1	6
miR-6500-3p	A_25_P00017878	0.878	0.292	2.543	0.06	6
miR-759	A_25_P00015466	0.930	0.262	-2.663	0.06	6

Table S3. Circadian characteristics of the miRNA expression profiles obtained from cosine-fitting function in a period of 28 hours for MDA-MB-231.

miRNA	agilent-code	cosine correlation	period	phase	amplitude	cluster
miR-3674	A_25_P00016190	0.856	0.238	1.670	0.11	1
miR-3910	A_25_P00016263	0.841	0.262	1.549	0.06	1
miR-4300	A_25_P00015719	0.832	0.296	0.634	0.09	1
miR-4654	A_25_P00016684	0.844	0.232	1.540	0.09	1
miR-4697-5p	A_25_P00016631	0.875	0.251	1.603	0.1	1
miR-4724-3p	A_25_P00016409	0.953	0.300	1.758	0.07	1
miR-503-5p	A_25_P00010657	0.913	0.283	1.904	0.07	1
miR-544b	A_25_P00015881	0.934	0.278	1.400	0.06	1
miR-548b-3p	A_25_P00010124	0.883	0.226	-3.142	0.07	1
miR-5583-5p	A_25_P00017479	0.871	0.233	1.240	0.14	1
miR-5688	A_25_P00017617	0.822	0.294	1.647	0.05	1
miR-6515-5p	A_25_P00017867	0.835	0.242	1.629	0.06	1
let-7b-3p	A_25_P00013118	0.957	0.278	0.435	0.1	2
miR-124-5p	A_25_P00013389	0.857	0.259	0.552	0.07	2
miR-142-3p	A_25_P00013937	0.827	0.230	1.382	0.05	2
miR-150-5p	A_25_P00010490	0.849	0.257	1.395	0.05	2
miR-23a-5p	A_25_P00013182	0.897	0.229	0.697	0.12	2
miR-28-3p	A_25_P00012007	0.886	0.279	0.669	0.16	2
miR-3195	A_25_P00015499	0.921	0.228	1.634	0.05	2
miR-325	A_25_P00010180	0.862	0.299	0.498	0.12	2
miR-3656	A_25_P00016105	0.906	0.280	0.423	0.06	2
miR-376a-2-5p	A_25_P00017745	0.866	0.260	0.806	0.09	2
miR-4252	A_25_P00015871	0.896	0.255	0.965	0.1	2
miR-4467	A_25_P00016337	0.942	0.295	-0.013	0.09	2
miR-4693-3p	A_25_P00016299	0.837	0.240	1.195	0.07	2
miR-4786-3p	A_25_P00016918	0.845	0.241	1.000	0.05	2
miR-520a-3p	A_25_P00014113	0.857	0.245	1.089	0.05	2
miR-611	A_25_P00010903	0.853	0.272	0.468	0.11	2
miR-615-5p	A_25_P00012787	0.825	0.286	0.357	0.14	2
miR-658	A_25_P00011946	0.887	0.239	0.757	0.09	2
dmr_316		0.951	0.271	-0.389	0.06	3
mr_1		0.919	0.290	-0.184	0.13	3
miR-1-3p	A_25_P00012150	0.982	0.287	-0.624	0.09	3
miR-101-5p	A_25_P00013250	0.842	0.272	-0.858	0.09	3
miR-1237-5p	A_25_P00017824	0.950	0.234	0.600	0.09	3
miR-182-3p	A_25_P00010966	0.925	0.273	0.045	0.06	3
miR-183-3p	A_25_P00013323	0.858	0.248	0.523	0.14	3
miR-185-3p	A_25_P00013457	0.952	0.297	-1.154	0.18	3
miR-1976	A_25_P00015365	0.910	0.277	-1.279	0.06	3

miR-219b-5p	A_25_P00016753	0.911	0.244	0.464	0.08	3
miR-2276-3p	A_25_P00015447	0.948	0.294	-0.442	0.08	3
miR-2277-3p	A_25_P00015459	0.969	0.274	-0.013	0.1	3
miR-23a-5p	A_25_P00013181	0.941	0.290	-0.847	0.11	3
miR-25-5p	A_25_P00013191	0.827	0.236	-0.139	0.11	3
miR-2681-3p	A_25_P00017021	0.964	0.280	0.119	0.07	3
miR-3120-3p	A_25_P00015853	0.857	0.297	-0.398	0.07	3
miR-3171	A_25_P00015735	0.946	0.249	0.068	0.08	3
miR-3194-3p	A_25_P00016421	0.878	0.272	-0.491	0.05	3
miR-33b-3p	A_25_P00013651	0.957	0.295	-0.861	0.05	3
miR-376c-3p	A_25_P00012316	0.889	0.299	-0.232	0.07	3
miR-3974	A_25_P00016911	0.874	0.282	-0.255	0.07	3
miR-4659b-3p	A_25_P00016492	0.874	0.263	0.039	0.12	3
miR-4744	A_25_P00016662	0.917	0.269	-0.427	0.07	3
miR-491-3p	A_25_P00012485	0.832	0.228	0.747	0.08	3
miR-499b-5p	A_25_P00017168	0.930	0.269	-1.066	0.08	3
miR-502-3p	A_25_P00012647	0.924	0.273	-0.442	0.07	3
miR-5093	A_25_P00017613	0.827	0.274	0.133	0.08	3
miR-541-5p	A_25_P00013667	0.886	0.276	-0.136	0.1	3
miR-548h-5p	A_25_P00015208	0.873	0.266	-0.006	0.06	3
miR-564	A_25_P00010786	0.893	0.282	-0.112	0.07	3
miR-5683	A_25_P00017565	0.885	0.230	0.964	0.09	3
miR-5685	A_25_P00017506	0.923	0.236	0.169	0.06	3
miR-5686	A_25_P00017376	0.833	0.290	-1.201	0.11	3
miR-573	A_25_P00011618	0.921	0.252	0.238	0.05	3
miR-607	A_25_P00010935	0.863	0.235	0.534	0.11	3
miR-6501-3p	A_25_P00017756	0.840	0.268	-0.938	0.16	3
miR-892b	A_25_P00012942	0.872	0.279	-0.093	0.1	3
miR-940	A_25_P00013090	0.913	0.283	-0.254	0.15	3
miR-1204	A_25_P00015082	0.923	0.275	-1.211	0.08	4
miR-1258	A_25_P00015167	0.950	0.254	-1.360	0.12	4
miR-1273a	A_25_P00015203	0.914	0.270	-1.739	0.07	4
miR-141-5p	A_25_P00013413	0.875	0.299	-1.577	0.08	4
miR-187-5p	A_25_P00013327	0.824	0.261	-1.404	0.13	4
miR-223-5p	A_25_P00013355	0.839	0.253	-1.416	0.22	4
miR-3157-5p	A_25_P00015563	0.826	0.231	-1.140	0.08	4
miR-33b-5p	A_25_P00012824	0.852	0.281	-1.534	0.07	4
miR-362-5p	A_25_P00013984	0.870	0.229	-1.220	0.06	4
miR-3660	A_25_P00016217	0.922	0.285	-1.845	0.07	4
miR-3925-5p	A_25_P00016056	0.824	0.259	-1.008	0.14	4
miR-4423-5p	A_25_P00016909	0.862	0.277	-1.804	0.07	4
miR-449b-5p	A_25_P00010776	0.932	0.293	-1.502	0.12	4
miR-4652-3p	A_25_P00017179	0.943	0.238	-0.728	0.07	4

miR-4725-3p	A_25_P00016713	0.862	0.258	-1.695	0.06	4
miR-4742-3p	A_25_P00016821	0.853	0.283	-1.502	0.08	4
miR-4787-3p	A_25_P00016945	0.944	0.262	-1.616	0.12	4
miR-4802-5p	A_25_P00016308	0.872	0.248	-0.937	0.1	4
miR-495-3p	A_25_P00012507	0.961	0.287	-1.767	0.1	4
miR-499a-3p	A_25_P00012626	0.843	0.240	-1.120	0.1	4
miR-505-3p	A_25_P00012654	0.875	0.238	-1.241	0.06	4
miR-512-3p	A_25_P00010710	0.924	0.272	-1.640	0.05	4
miR-567	A_25_P00010942	0.914	0.272	-1.688	0.07	4
miR-590-5p	A_25_P00014257	0.855	0.244	-1.025	0.1	4
miR-101-5p	A_25_P00014977	0.853	0.234	-1.844	0.13	5
miR-124-3p	A_25_P00014839	0.979	0.267	-2.116	0.06	5
miR-17-5p	A_25_P00011991	0.987	0.247	-2.122	0.17	5
miR-193b-3p	A_25_P00012512	0.848	0.277	-2.529	0.09	5
miR-196a-5p	A_25_P00012052	0.849	0.279	-2.288	0.08	5
miR-19b-2-5p	A_25_P00013164	0.828	0.248	-1.705	0.12	5
miR-30c-5p	A_25_P00013883	0.821	0.284	-2.177	0.17	5
miR-3119	A_25_P00015746	0.968	0.249	-1.737	0.15	5
miR-3675-5p	A_25_P00016157	0.895	0.255	-2.528	0.05	5
miR-3689f	A_25_P00017126	0.932	0.258	-2.472	0.08	5
miR-4308	A_25_P00015502	0.857	0.287	-2.148	0.06	5
miR-452-3p	A_25_P00013583	0.954	0.231	-1.965	0.07	5
miR-4520-5p	A_25_P00017309	0.847	0.265	-2.470	0.06	5
miR-4531	A_25_P00017111	0.828	0.258	-2.300	0.07	5
miR-4684-5p	A_25_P00017088	0.823	0.274	-1.832	0.1	5
miR-4700-3p	A_25_P00016640	0.922	0.251	-1.639	0.06	5
miR-4768-3p	A_25_P00016594	0.979	0.282	-2.164	0.08	5
miR-486-3p	A_25_P00012469	0.846	0.272	-2.130	0.07	5
miR-532-5p	A_25_P00014179	0.911	0.284	-2.879	0.07	5
miR-548at-5p	A_25_P00017425	0.861	0.266	-2.005	0.14	5
miR-5584-5p	A_25_P00017674	0.946	0.243	-1.872	0.05	5
miR-571	A_25_P00010633	0.933	0.274	-2.086	0.1	5
miR-6081	A_25_P00017906	0.868	0.252	-1.631	0.09	5
miR-6128	A_25_P00017983	0.912	0.261	-1.958	0.06	5
miR-6165	A_25_P00017841	0.962	0.267	-2.139	0.08	5
miR-619-3p	A_25_P00011293	0.891	0.260	-2.039	0.12	5
miR-769-3p	A_25_P00011231	0.915	0.229	-1.591	0.12	5
miR-942-5p	A_25_P00013098	0.856	0.261	-1.641	0.1	5
miR-106b-5p	A_25_P00010434	0.828	0.260	-2.722	0.29	6
miR-130b-5p	A_25_P00013503	0.946	0.283	-2.981	0.09	6
miR-18a-3p	A_25_P00013155	0.848	0.291	3.142	0.16	6
miR-196b-5p	A_25_P00012412	0.821	0.262	-2.662	0.06	6
miR-21-3p	A_25_P00013173	0.931	0.291	3.021	0.12	6

miR-23c	A_25_P00016198	0.837	0.227	-2.583	0.06	6
miR-302b-3p	A_25_P00010618	0.823	0.235	-2.131	0.06	6
miR-302d-3p	A_25_P00010163	0.902	0.271	-2.998	0.1	6
miR-3074-5p	A_25_P00016835	0.866	0.230	-2.038	0.06	6
miR-3622a-5p	A_25_P00016140	0.854	0.251	-2.221	0.13	6
miR-3714	A_25_P00016015	0.922	0.275	-3.142	0.05	6
miR-371a-3p	A_25_P00013992	0.921	0.239	-2.155	0.07	6
miR-423-3p	A_25_P00012422	0.844	0.286	2.788	0.11	6
miR-4325	A_25_P00015808	0.835	0.299	-3.018	0.06	6
miR-455-3p	A_25_P00012698	0.839	0.227	-2.203	0.08	6
miR-4667-5p	A_25_P00017004	0.914	0.254	-1.953	0.05	6
miR-4704-5p	A_25_P00016353	0.863	0.264	2.917	0.06	6
miR-4715-3p	A_25_P00016596	0.828	0.233	-1.833	0.05	6
miR-548as-3p	A_25_P00017374	0.926	0.292	2.495	0.05	6
miR-548ay-3p	A_25_P00017966	0.862	0.250	-3.027	0.08	6
miR-5582-3p	A_25_P00017509	0.937	0.231	-2.320	0.07	6
miR-636	A_25_P00012829	0.985	0.285	2.307	0.07	6
miR-655-3p	A_25_P00011229	0.897	0.262	-2.210	0.11	6
miR-885-3p	A_25_P00012992	0.951	0.239	-1.945	0.13	6
miR-92a-2-5p	A_25_P00013230	0.823	0.243	-2.642	0.07	6
miR-92a-3p	A_25_P00012031	0.848	0.297	2.828	0.25	6
miR-938	A_25_P00013083	0.868	0.283	-2.982	0.05	6

Table S4. Enriched pathways from 343 targets of miR-17-5p.

Nº	Pathway identifier	Pathway name	Number of genes found	Total genes	pValue	FDR	Genes found
1	R-HSA-69236	G1 Phase	12	42	1.59E-08	9.90E-06	CCL1;E2F1;PPP2R1A;CDKN1A;CCND1;E2F3;RBL2;CCND2;RBL1;RB1;RPS27A
2	R-HSA-69231	Cyclin D associated events in G1	12	42	1.59E-08	9.90E-06	CCL1;E2F1;PPP2R1A;CDKN1A;CCND1;E2F3;RBL2;CCND2;RBL1;RB1;RPS27A
3	R-HSA-3247509	Chromatin modifying enzymes	24	247	1.73E-06	5.40E-04	HIST2H2AA3;HIST4H4;HIST1H4L;HIST2H2AA4;HIST1H4K;KMT2A;HIST1H2AG;TAF9B;MORF4L2;TRRAP;HIST2H4A;HIST2H4B;HIST1H4A;HIST1H4B;HIST1H4E;HIST1H4F;HIST3H2A;HIST1H4C;HIST1H4D;HIST1H4I;HIST1H4J;CHD4;HIST1H4H;KAT2A;HIST2H3A;ELP2;KANSL1;HDAC10;EHMT2;HIST2H3C;HIST2H3D;CCND1;ATXN7;HIST1H2AI;HIST1H2AK;KDM4A;HIST1H2AM;HIST1H2AL
4	R-HSA-4839726	Chromatin organization	24	247	1.73E-06	5.40E-04	HIST2H2AA3;HIST4H4;HIST1H4L;HIST2H2AA4;HIST1H4K;KMT2A;HIST1H2AG;TAF9B;MORF4L2;TRRAP;HIST2H4A;HIST2H4B;HIST1H4A;HIST1H4B;HIST1H4E;HIST1H4F;HIST3H2A;HIST1H4C;HIST1H4D;HIST1H4I;HIST1H4J;CHD4;HIST1H4H;KAT2A;HIST2H3A;ELP2;KANSL1;HDAC10;EHMT2;HIST2H3C;HIST2H3D;CCND1;ATXN7;HIST1H2AI;HIST1H2AK;KDM4A;HIST1H2AM;HIST1H2AL
5	R-HSA-2173789	TGF-beta receptor signaling activates SMADs	9	36	2.93E-06	6.85E-04	PPP1CA;CBL;TGFBR2;SMAD4;SMAD3;PPP1R15A;RPS27A
6	R-HSA-3214847	HATs acetylate histones	15	110	3.30E-06	6.85E-04	HIST2H2AA3;HIST4H4;HIST1H4L;HIST2H2AA4;HIST1H4K;HIST1H2AG;TAF9B;MORF4L2;TRRAP;HIST2H4A;HIST2H4B;HIST1H4A;HIST1H4B;HIST1H4E;HIST1H4F;HIST3H2A;HIST1H4C;HIST1H4D;HIST1H4I;HIST1H4J;HIST1H4H;HIST2H3A;KAT2A;ELP2;KANSL1;HIST2H3C;HIST2H3D;ATXN7;HIST1H2AI;HIST1H2AK;HIST1H2AM;HIST1H2AL
7	R-HSA-170834	Signaling by TGF-beta Receptor Complex	13	84	3.92E-06	6.97E-04	MEN1;PPP1CA;CBL;RBL1;TGFBR2;SMAD4;SMAD3;PPP1R15A;MYC;RPS27A
8	R-HSA-2173788	Downregulation of TGF-beta receptor signaling	7	28	3.72E-05	0.004285289	PPP1CA;TGFBR2;SMAD3;PPP1R15A;RPS27A
9	R-HSA-157118	Signaling by NOTCH	16	153	3.97E-05	0.004285289	KAT2A;E2F1;NOTCH2;CCND1;E2F3;HDAC10;AGO1;MYC;MDK;RPS27A
10	R-HSA-3214815	HDACs deacetylate histones	10	63	4.12E-05	0.004285289	HIST2H2AA3;HIST4H4;HIST1H4L;HIST2H2AA4;HIST1H4K;HIST1H2AG;HIST2H4A;HIST2H4B;HIST1H4A;HIST1H4B;HIST1H4E;HIST1H4F;HIST3H2A;HIST1H4C;HIST1H4D;HIST1H4I;HIST1H4J;CHD4;HIST1H4H;HIST2H3A;HDAC10;HIST2H3C;HIST2H3D;HIST1H2AI;HIST1H2AK;HIST1H2AM;HIST1H2AL

11	R-HSA-2173796	SMAD2/SMAD3:SMAD4 heterotrimer regulates transcription	8	39	4.19E-05	0.004285289	MEN1;RBL1;SMAD4;SMAD3;MYC;RPS27A
12	R-HSA-3214858	RMTs methylate histone arginines	9	51	4.46E-05	0.004285289	HIST2H2AA3;HIST4H4;HIST1H4L;HIST2H2AA4;HIST1H4K;HIST1H2AG;HIST2H4A;HIST2H4B;HIST1H4A;HIST1H4B;HIST1H4E;HIST1H4F;HIST3H2A;HIST1H4C;HIST1H4D;HIST1H4I;HIST1H4J;HIST1H4H;HIST2H3A;HIST2H3C;HIST2H3D;CCND1;HIST1H2AI;HIST1H2AK;HIST1H2AM;HIST1H2AL
13	R-HSA-1912408	Pre-NOTCH Transcription and Translation	9	52	5.17E-05	0.004603601	KAT2A;E2F1;NOTCH2;CCND1;E2F3;AGO1
14	R-HSA-201722	Formation of the beta-catenin:TCF transactivating complex	10	66	6.03E-05	0.005007399	HIST2H2AA3;HIST4H4;HIST1H4L;HIST2H2AA4;HIST1H4K;HIST1H2AG;TRRAP;HIST2H4A;HIST2H4B;MEN1;HIST1H4A;HIST1H4B;HIST1H4E;HIST1H4F;HIST1H4C;HIST1H4D;HIST1H4I;TCF3;MYC;HIST1H4J;HIST1H4H;HIST2H3A;HIST2H3C;HIST2H3D;HIST1H2AI;HIST1H2AK;HIST1H2AL
15	R-HSA-453279	Mitotic G1-G1/S phases	15	143	6.65E-05	0.005109995	CCL1;E2F1;PPP2R1A;E2F3;RBL2;RBL1;RB1;WEE1;CDKN1A;CCND1;CCND2;MYC;RPS27A
16	R-HSA-3214842	HDMs demethylate histones	7	31	7.00E-05	0.005109995	HIST2H3A;HIST1H4L;HIST4H4;HIST1H4K;HIST2H4A;HIST2H3C;HIST2H4B;HIST2H3D;HIST1H4A;HIST1H4B;HIST1H4E;HIST1H4F;HIST1H4C;HIST1H4D;KDM4A;HIST1H4I;HIST1H4J;HIST1H4H
17	R-HSA-5689901	Metalloprotease DUBs	7	32	8.51E-05	0.00587264	HIST2H2AA3;HIST2H2AA4;HIST1H2AG;HIST1H2AI;HIST3H2A;HIST1H2AK;HIST1H2AM;HIST1H2AL;RPS27A
18	R-HSA-3700989	Transcriptional Regulation by TP53	31	476	1.44E-04	0.00812336	CCL1;E2F1;PPP2R1A;RBL2;STK11;RBL1;COX7B;TAF9B;EHMT2;PTEN;NOC2L;GPI;PDPK1;CDKN1A;TNFRSF10B;MT-CO2;TSC2;AGO1;RPS27A;CHD4;ERCC2
19	R-HSA-2559582	Senescence-Associated Secretory Phenotype (SASP)	11	89	1.56E-04	0.00812336	HIST2H2AA3;HIST4H4;HIST1H4L;HIST2H2AA4;HIST1H4K;HIST1H2AG;IL-8;HIST2H4A;HIST2H4B;HIST1H4A;HIST1H4B;HIST1H4E;HIST1H4F;HIST1H4C;HIST1H4D;HIST1H4I;HIST1H4J;RPS27A;HIST1H4H;HIST2H3A;EHMT2;UBE2C;HIST2H3C;HIST2H3D;CDKN1A;HIST1H2AI;HIST1H2AK;HIST1H2AL
20	R-HSA-937039	IRAK1 recruits IKK complex	5	16	1.77E-04	0.00812336	IRAK1;PELI1;RPS27A
21	R-HSA-975144	IRAK1 recruits IKK complex upon TLR7/8 or 9 stimulation	5	16	1.77E-04	0.00812336	IRAK1;PELI1;RPS27A
22	R-HSA-1912422	Pre-NOTCH Expression and Processing	10	76	1.88E-04	0.008179777	KAT2A;E2F1;NOTCH2;CCND1;E2F3;AGO1
23	R-HSA-2559583	Cellular Senescence	17	194	1.90E-04	0.008179777	E2F1;HIST2H2AA3;HIST4H4;HIST1H4L;HIST2H2AA4;E2F3;HIST1H4K;HIST1H2AG;IL-8;HIST2H4A;HIST2H4B;HIST1H4A;HIST1H4B;AGO1;HIST1H4E;

							HIST1H4F;HIST1H4C;HIST1H4D;HIST1H4I;HIST1H4J;RPS27A;HIST1H4H;HIST2H3A;RB1;UBE2C;EHMT2;HIST2H3C;HIST2H3D;CDKN1A;HIST1H2AI;MAPK9;HIST1H2AK;HIST1H2AL
24	R-HSA-73854	RNA Polymerase I Promoter Clearance	10	83	3.74E-04	0.015324076	CCL1;HIST2H2AA3;HIST4H4;HIST1H4L;HIST2H2AA4;HIST1H4K;HIST1H2AG;HIST2H4A;HIST2H4B;HIST1H4A;HIST1H4B;HIST1H4E;HIST1H4F;HIST1H4C;HIST1H4D;HIST1H4I;HIST1H4J;CHD4;HIST1H4H;ERCC2;KAT2A;HIST2H3A;EHMT2;HIST2H3C;HIST2H3D;HIST1H2AI;HIST1H2AK;HIST1H2AL
25	R-HSA-6804116	TP53 Regulates Transcription of Genes Involved in G1 Cell Cycle Arrest	5	19	3.86E-04	0.015445318	E2F1;CDKN1A
26	R-HSA-2173793	Transcriptional activity of SMAD2/SMAD3:SMAD4 heterotrimer	8	55	4.25E-04	0.015737346	MEN1;RBL1;SMAD4;SMAD3;MYC;RPS27A
27	R-HSA-5675482	Regulation of necroptotic cell death	5	20	4.86E-04	0.017182244	TNFRSF10B;XIAP;RPS27A
28	R-HSA-73864	RNA Polymerase I Transcription	10	86	4.91E-04	0.017182244	CCL1;HIST2H2AA3;HIST4H4;HIST1H4L;HIST2H2AA4;HIST1H4K;HIST1H2AG;HIST2H4A;HIST2H4B;HIST1H4A;HIST1H4B;HIST1H4E;HIST1H4F;HIST1H4C;HIST1H4D;HIST1H4I;HIST1H4J;CHD4;HIST1H4H;ERCC2;KAT2A;HIST2H3A;EHMT2;HIST2H3C;HIST2H3D;HIST1H2AI;HIST1H2AK;HIST1H2AL
29	R-HSA-69202	Cyclin E associated events during G1/S transition	9	73	6.17E-04	0.020988318	CCL1;CDKN1A;CCND1;RB1;MYC;RPS27A;WEE1
30	R-HSA-2559580	Oxidative Stress Induced Senescence	11	109	8.32E-04	0.027454642	E2F1;HIST2H2AA3;HIST4H4;HIST1H4L;HIST2H2AA4;E2F3;HIST1H4K;HIST1H2AG;HIST2H4A;HIST2H4B;HIST1H4A;HIST1H4B;AGO1;HIST1H4E;HIST1H4F;HIST1H4C;HIST1H4D;HIST1H4I;HIST1H4J;RPS27A;HIST1H4H;HIST2H3A;HIST2H3C;HIST2H3D;HIST1H2AI;MAPK9;HIST1H2AK;HIST1H2AL
31	R-HSA-8866911	TFAP2 (AP-2) family regulates transcription of cell cycle factors	3	6	9.77E-04	0.03027922	CDKN1A;MYC
32	R-HSA-69895	Transcriptional activation of cell cycle inhibitor p21	3	6	9.77E-04	0.03027922	CDKN1A
33	R-HSA-69560	Transcriptional activation of p53 responsive genes	3	6	9.77E-04	0.03027922	CDKN1A
34	R-HSA-5689880	Ub-specific processing proteases	16	206	0.001039758	0.030958242	KAT2A;HIST2H2AA3;HIST2H2AA4;HIST1H2AG;TAF9B;SMAD4;SMAD3;TRRAP;PTEN;ATXN7;HIST1H2AI;HIST3H2A;HIST1H2AK;HIST1H2AM;HIST1H2AL;MYC;RNF146;RPS27A
35	R-HSA-427389	ERCC6 (CSB) and EHMT2 (G9a) positively regulate rRNA expression	7	49	0.001067526	0.030958242	HIST2H2AA3;HIST4H4;HIST1H4L;HIST2H2AA4;HIST1H4K;HIST1H2AG;HIST2H4A;HIST2H4B;HIST1H4A;HIST1H4B;HIST1H4E;HIST1H4F;HIST1H4C;HIST1H4D;HIST1H4I;HIST1H4J;CHD4;HIS

							T1H4H;HIST2H3A;EHMT2;HIST2H3C;HIST2H3D;HIST1H2AI;HIST1H2AK;HIST1H2AL
36	R-HSA-912631	Regulation of signaling by CBL	5	24	0.001092732	0.031689229	CBL;PIK3CA;YES1;RPS27A
37	R-HSA-69298	Association of licensing factors with the pre-replicative complex	4	15	0.001421808	0.037989713	E2F1;E2F3;RPS27A
38	R-HSA-5637815	Signaling by Ligand-Responsive EGFR Variants in Cancer	6	38	0.001461143	0.037989713	HSP90AA1;CBL;GAB1;PIK3CA;RPS27A
39	R-HSA-1236382	Constitutive Signaling by Ligand-Responsive EGFR Cancer Variants	6	38	0.001461143	0.037989713	HSP90AA1;CBL;GAB1;PIK3CA;RPS27A
40	R-HSA-1643713	Signaling by EGFR in Cancer	6	38	0.001461143	0.037989713	HSP90AA1;CBL;GAB1;PIK3CA;RPS27A
41	R-HSA-69278	Cell Cycle, Mitotic	30	526	0.001525499	0.038137477	CCL1;E2F1;HIST2H2AA3;HIST4H4;HIST1H4L;HIST2H2AA4;E2F3;HIST1H4K;HIST1H2AG;CETN2;PTTG1;HIST2H4A;HIST2H4B;OFD1;HIST1H4A;HIST1H4B;HIST1H4E;HIST1H4F;HIST1H4C;HIST1H4D;HIST1H4I;HIST1H4J;MYC;RPS27A;TUBB4B;HIST1H4H;HIST2H3A;PPP2R1A;HSP90AA1;RBL2;RBL1;OPTN;RB1;UBE2C;LPIN1;WEE1;HIST2H3C;HIST2H3D;PPP1CA;CDKN1A;CCND1;CCND2;HIST1H2AI;CNEP1R1;HIST1H2AK;HIST1H2AL
42	R-HSA-201681	TCF dependent signaling in response to WNT	16	215	0.00160079	0.038418951	HIST2H2AA3;HIST4H4;HIST1H4L;HIST2H2AA4;HIST1H4K;XIAP;HIST1H2AG;SOX4;TRRAP;HIST2H4A;HIST2H4B;MEN1;HIST1H4A;HIST1H4B;HIST1H4E;HIST1H4F;HIST1H4C;HIST1H4D;HIST1H4I;TCF3;HIST1H4J;MYC;RNF146;RPS27A;HIST1H4H;HIST2H3A;PPP2R1A;HIST2H3C;HIST2H3D;HIST1H2AI;HIST1H2AK;HIST1H2AL
43	R-HSA-69206	G1/S Transition	11	119	0.001658811	0.039811459	CCL1;E2F1;PPP2R1A;CDKN1A;CCND1;RB1;MYC;RPS27A;WEE1
44	R-HSA-209543	p75NTR recruits signalling complexes	4	16	0.001795865	0.041956746	IRAK1;RPS27A
45	R-HSA-5218859	Regulated Necrosis	5	27	0.001824206	0.041956746	TNFRSF10B;XIAP;RPS27A
46	R-HSA-5213460	RIPK1-mediated regulated necrosis	5	27	0.001824206	0.041956746	TNFRSF10B;XIAP;RPS27A
47	R-HSA-195721	Signaling by Wnt	21	329	0.002123731	0.044663082	HIST2H2AA3;HIST4H4;HIST1H4L;HIST2H2AA4;HIST1H4K;XIAP;HIST1H2AG;SOX4;TRRAP;HIST2H4A;HIST2H4B;MEN1;HIST1H4A;HIST1H4B;AGO1;HIST1H4E;HIST1H4F;HIST1H4C;HIST1H4D;HIST1H4I;TCF3;HIST1H4J;MYC;RNF146;RPS27A;HIST1H4H;HIST2H3A;PPP2R1A;HIST2H3C;HIST2H3D;PRICKLE1;GNB1;HIST1H2AI;HIST1H2AK;HIST1H2AL
48	R-HSA-453274	Mitotic G2-G2/M phases	15	201	0.002155993	0.044663082	CCL1;OFD1;E2F1;PPP2R1A;CDKN1A;E2F3;HSP90AA1;CETN2;OPTN;RPS27A;WEE1;TUBB4B
49	R-HSA-1295596	Spry regulation of FGF signaling	4	17	0.002233154	0.044663082	PPP2R1A;CBL;RPS27A
50	R-HSA-69656	Cyclin A:Cdk2-associated events at S phase entry	8	74	0.002740151	0.049552658	CCL1;CDKN1A;CCND1;RB1;MYC;RPS27A;WEE1

51	R-HSA-400253	Circadian Clock	9	91	0.002752925	0.049552658	MEF2D;PPP1CA;NPAS2;PER1;RPS27A
52	R-HSA-975871	MyD88 cascade initiated on plasma membrane	9	91	0.002752925	0.049552658	IRAK1;PPP2R1A;APP;PELI1;MAP3K8;MAPK9;RPS27A
53	R-HSA-168176	Toll Like Receptor 5 (TLR5) Cascade	9	91	0.002752925	0.049552658	IRAK1;PPP2R1A;APP;PELI1;MAP3K8;MAPK9;RPS27A
54	R-HSA-168142	Toll Like Receptor 10 (TLR10) Cascade	9	91	0.002752925	0.049552658	IRAK1;PPP2R1A;APP;PELI1;MAP3K8;MAPK9;RPS27A
55	R-HSA-2565942	Regulation of PLK1 Activity at G2/M Transition	9	92	0.002956405	0.052269774	OFD1;PPP2R1A;HSP90AA1;CETN2;OPTN;RPS27A;TUBB4B
56	R-HSA-3769402	Deactivation of the beta-catenin transactivating complex	6	44	0.003012032	0.052269774	MEN1;XIAP;SOX4;TCF3;RPS27A
57	R-HSA-3304349	Loss of Function of SMAD2/3 in Cancer	3	9	0.003074693	0.052269774	TGFBR2;SMAD4;SMAD3
58	R-HSA-3315487	SMAD2/3 MH2 Domain Mutants in Cancer	3	9	0.003074693	0.052269774	TGFBR2;SMAD4;SMAD3
59	R-HSA-4411364	Binding of TCF/LEF:CTNNB1 to target gene promoters	3	9	0.003074693	0.052269774	TCF3;MYC
60	R-HSA-975138	TRAF6 mediated induction of NFkB and MAP kinases upon TLR7/8 or 9 activation	9	93	0.003171581	0.053082475	IRAK1;PPP2R1A;APP;PELI1;MAP3K8;MAPK9;RPS27A
61	R-HSA-193639	p75NTR signals via NF-kB	4	19	0.003317655	0.053082475	IRAK1;RPS27A
62	R-HSA-73777	RNA Polymerase I Chain Elongation	7	61	0.003599774	0.05635569	CCL1;HIST2H2AA3;HIST4H4;HIST1H4L;HIST2H2AA4;HIST1H4K;HIST1H2AG;HIST2H4A;HIST2H4B;HIST1H4A;HIST1H4B;HIST1H4E;HIST1H4F;HIST1H4C;HIST1H4D;HIST1H4I;HIST1H4J;HIST1H4H;ERCC2;HIST2H3A;HIST2H3C;HIST2H3D;HIST1H2AI;HIST1H2AK;HIST1H2AL
63	R-HSA-975155	MyD88 dependent cascade initiated on endosome	9	95	0.003638866	0.05635569	IRAK1;PPP2R1A;APP;PELI1;MAP3K8;MAPK9;RPS27A
64	R-HSA-168181	Toll Like Receptor 7/8 (TLR7/8) Cascade	9	95	0.003638866	0.05635569	IRAK1;PPP2R1A;APP;PELI1;MAP3K8;MAPK9;RPS27A
65	R-HSA-380994	ATF4 activates genes	5	32	0.003757046	0.05635569	ATF3;ASNS;IL-8
66	R-HSA-3304351	Signaling by TGF-beta Receptor Complex in Cancer	3	10	0.004121177	0.061817651	TGFBR2;SMAD4;SMAD3
67	R-HSA-3311021	SMAD4 MH2 Domain Mutants in Cancer	2	3	0.004202011	0.062868764	SMAD4;SMAD3
68	R-HSA-3304347	Loss of Function of SMAD4 in Cancer	2	3	0.004202011	0.062868764	SMAD4;SMAD3
69	R-HSA-168138	Toll Like Receptor 9 (TLR9) Cascade	9	98	0.004439226	0.062868764	IRAK1;PPP2R1A;APP;PELI1;MAP3K8;MAPK9;RPS27A
70	R-HSA-109581	Apoptosis	13	176	0.004509212	0.062868764	E2F1;TJP1;TNFRSF10B;XIAP;BCL2;VIM;BIM;PIGS;DAPK3;BCL2L

							11;RPS27A;ARHGAP10
71	R-HSA-446652	Interleukin-1 signaling	6	48	0.004576186	0.062868764	IRAK1;PELI1;MAP3K8;RPS27A
72	R-HSA-6791312	TP53 Regulates Transcription of Cell Cycle Genes	7	64	0.004654248	0.062868764	E2F1;CDKN1A;RBL2;RBL1
73	R-HSA-427413	NoRC negatively regulates rRNA expression	8	81	0.004682595	0.062868764	CCL1;HIST2H2AA3;HIST4H4;HIST1H4L;HIST2H2AA4;HIST1H4K;HIST1H2AG;HIST2H4A;HIST2H4B;HIST1H4A;HIST1H4B;HIST1H4E;HIST1H4F;HIST1H4C;HIST1H4D;HIST1H4I;HIST1H4J;BAZ2A;HIST1H4H;ERCC2;HIST2H3A;HIST2H3C;HIST2H3D;HIST1H2AI;HIST1H2AK;HIST1H2AL
74	R-HSA-5250913	Positive epigenetic regulation of rRNA expression	8	81	0.004682595	0.062868764	HIST2H2AA3;HIST4H4;HIST1H4L;HIST2H2AA4;HIST1H4K;HIST1H2AG;HIST2H4A;HIST2H4B;HIST1H4A;HIST1H4B;HIST1H4E;HIST1H4F;HIST1H4C;HIST1H4D;HIST1H4I;HIST1H4J;CHD4;HIST1H4H;KAT2A;HIST2H3A;EHMT2;HIST2H3C;HIST2H3D;HIST1H2AI;HIST1H2AK;HIST1H2AL
75	R-HSA-5688426	Deubiquitination	18	285	0.00477305	0.062868764	KAT2A;HIST2H2AA3;HIST2H2AA4;HIST1H2AG;TGFBR2;TAF9B;SMAD4;SMAD3;TRRAP;PTEN;ATXN7;HIST1H2AI;HIST3H2A;HIST1H2AK;CAP1;HIST1H2AM;HIST1H2AL;MYC;RNF146;RPS27A
76	R-HSA-73728	RNA Polymerase I Promoter Opening	5	34	0.004836059	0.062868764	HIST2H3A;HIST2H2AA3;HIST2H2AA4;HIST1H4L;HIST4H4;HIST1H4K;HIST1H2AG;HIST2H4A;HIST2H3C;HIST2H4B;HIST2H3D;HIST1H4A;HIST1H4B;HIST1H4E;HIST1H2AI;HIST1H4F;HIST1H4C;HIST1H2AK;HIST1H4D;HIST1H4I;HIST1H2AL;HIST1H4J;HIST1H4H
77	R-HSA-8849469	PTK6 Regulates RTKs and Their Effectors AKT1 and DOK1	3	11	0.005359972	0.069679641	CBL;RPS27A
78	R-HSA-2979096	NOTCH2 Activation and Transmission of Signal to the Nucleus	4	22	0.005541623	0.072041099	NOTCH2;MDK;RPS27A
79	R-HSA-212165	Epigenetic regulation of gene expression	11	140	0.005571449	0.072428838	CCL1;HIST2H2AA3;HIST4H4;HIST1H4L;HIST2H2AA4;HIST1H4K;HIST1H2AG;HIST2H4A;HIST2H4B;HIST1H4A;HIST1H4B;HIST1H4E;HIST1H4F;HIST1H4C;HIST1H4D;HIST1H4I;HIST1H4J;BAZ2A;CHD4;HIST1H4H;ERCC2;KAT2A;HIST2H3A;EHMT2;HIST2H3C;HIST2H3D;HIST1H2AI;HIST1H2AK;HIST1H2AL
80	R-HSA-5357801	Programmed Cell Death	13	184	0.006441873	0.082077289	E2F1;TJP1;TNFRSF10B;XIAP;BCL2;VIM;BIM;PIGS;DAPK3;BCL2L11;RPS27A;ARHGAP10
81	R-HSA-504046	RNA Polymerase I, RNA Polymerase III, and Mitochondrial Transcription	10	124	0.006755601	0.082077289	CCL1;HIST2H2AA3;HIST4H4;HIST1H4L;HIST2H2AA4;HIST1H4K;HIST1H2AG;HIST2H4A;HIST2H4B;HIST1H4A;HIST1H4B;HIST1H4E;HIST1H4F;HIST1H4C;HIST1H4D;HIST1H4I;HIST1H4J;CHD4;HIST1H4H;ERCC2;KAT2A;HIST2H3A;EHMT2;HIST2H3C;HIST2H3D;HIST1H2AI;HIST1H2AK;HIST1H2AL

82	R-HSA-113501	Inhibition of replication initiation of damaged DNA by RB1/E2F1	3	12	0.006799982	0.082077289	E2F1;PPP2R1A;RB1
83	R-HSA-5687128	MAPK6/MAPK4 signaling	9	105	0.006839774	0.082077289	HSP27;NCOA3;AGO1;MMP2;MYC;RPS27A
84	R-HSA-5334118	DNA methylation	5	37	0.006841886	0.082102628	HIST2H3A;HIST2H2AA3;HIST2H2AA4;HIST1H4L;HIST4H4;HIST1H4K;HIST1H2AG;HIST2H4A;HIST2H3C;HIST2H4B;HIST2H3D;HIST1H4A;HIST1H4B;HIST1H4E;HIST1H2AI;HIST1H4F;HIST1H4C;HIST1H2AK;HIST1H4D;HIST1H4I;HIST1H2AL;HIST1H4J;HIST1H4H
85	R-HSA-1640170	Cell Cycle	32	638	0.007280208	0.082223678	CCL1;E2F1;HIST2H2AA3;HIST4H4;HIST1H4L;HIST2H2AA4;E2F3;HIST1H4K;HIST1H2AG;CETN2;PTTG1;HIST2H4A;HIST2H4B;OFD1;HIST1H4A;HIST1H4B;HIST1H4E;HIST1H4F;HIST1H4C;HIST1H4D;HIST1H4I;HIST1H4J;MYC;RPS27A;TUBB4B;HIST1H4H;HIST2H3A;PPP2R1A;HSP90AA1;RBL2;RBL1;OPTN;RB1;UBE2C;LPIN1;WEE1;HIST2H3C;HIST2H3D;PPP1CA;CDKN1A;CCND1;CCND2;HIST1H2AI;CNEP1R1;HIST1H2AK;HIST1H2AL
86	R-HSA-450321	JNK (c-Jun kinases) phosphorylation and activation mediated by activated human TAK1	4	24	0.00747488	0.082223678	IRAK1;MAPK9;RPS27A
87	R-HSA-381042	PERK regulates gene expression	5	38	0.007623025	0.083258675	ATF3;ASNS;IL-8
88	R-HSA-166058	MyD88:Mal cascade initiated on plasma membrane	9	107	0.00768109	0.083258675	IRAK1;PPP2R1A;APP;PELI1;MAP3K8;MAPK9;RPS27A
89	R-HSA-168188	Toll Like Receptor TLR6:TLR2 Cascade	9	107	0.00768109	0.083258675	IRAK1;PPP2R1A;APP;PELI1;MAP3K8;MAPK9;RPS27A
90	R-HSA-166054	Activated TLR4 signalling	10	128	0.008325868	0.083258675	IRAK1;PPP2R1A;PELI1;APP;MAP3K8;MAPK9;CAP1;RPS27A
91	R-HSA-350054	Notch-HLH transcription pathway	3	13	0.008448702	0.084487023	KAT2A;NOTCH2
92	R-HSA-2559585	Oncogene Induced Senescence	5	39	0.008464165	0.084641645	E2F1;E2F3;AGO1;RPS27A
93	R-HSA-5250941	Negative epigenetic regulation of rRNA expression	8	90	0.008557998	0.085579979	CCL1;HIST2H2AA3;HIST4H4;HIST1H4L;HIST2H2AA4;HIST1H4K;HIST1H2AG;HIST2H4A;HIST2H4B;HIST1H4A;HIST1H4B;HIST1H4E;HIST1H4F;HIST1H4C;HIST1H4D;HIST1H4I;HIST1H4J;BAZ2A;HIST1H4H;ERCC2;HIST2H3A;HIST2H3C;HIST2H3D;HIST1H2AI;HIST1H2AK;HIST1H2AL
94	R-HSA-1227986	Signaling by ERBB2	6	55	0.008642057	0.086420573	HSP90AA1;GAB1;PIK3CA;YES1;RPS27A
95	R-HSA-168179	Toll Like Receptor TLR1:TLR2 Cascade	9	110	0.009088326	0.090883262	IRAK1;PPP2R1A;APP;PELI1;MAP3K8;MAPK9;RPS27A
96	R-HSA-181438	Toll Like Receptor 2 (TLR2) Cascade	9	110	0.009088326	0.090883262	IRAK1;PPP2R1A;APP;PELI1;MAP3K8;MAPK9;RPS27A
97	R-HSA-2122947	NOTCH1 Intracellular Domain Regulates Transcription	6	56	0.009383589	0.093015406	KAT2A;HDAC10;MYC;RPS27A

98	R-HSA-5637812	Signaling by EGFRvIII in Cancer	4	26	0.009806506	0.093015406	HSP90AA1;CBL;GAB1;PIK3CA
99	R-HSA-5637810	Constitutive Signaling by EGFRvIII	4	26	0.009806506	0.093015406	HSP90AA1;CBL;GAB1;PIK3CA
100	R-HSA-174490	Membrane binding and targetting of GAG proteins	3	14	0.010312321	0.093015406	TSG101;RPS27A
101	R-HSA-901042	Calnexin/calreticulin cycle	5	41	0.010335045	0.093015406	GANAB;DHX33;CANX;RPS27A
102	R-HSA-2173795	Downregulation of SMAD2/3:SMAD4 transcriptional activity	4	27	0.01113077	0.100176927	SMAD4;SMAD3;RPS27A
103	R-HSA-3214841	PKMTs methylate histone lysines	5	42	0.011368941	0.102320468	HIST2H3A;HIST1H4L;HIST4H4;HIST1H4K;KMT2A;HIST2H4A;EHMT2;HIST2H3C;HIST2H4B;HIST2H3D;HIST1H4A;HIST1H4B;HIST1H4E;HIST1H4F;HIST1H4C;HIST1H4D;HIST1H4I;HIST1H4J;HIST1H4H
104	R-HSA-69275	G2/M Transition	13	199	0.011798815	0.106189334	CCL1;OFD1;PPP2R1A;CDKN1A;HSP90AA1;CETN2;OPTN;RPS27A;WEE1;TUBB4B
105	R-HSA-209560	NF-kB is activated and signals survival	3	15	0.012395813	0.111562318	IRAK1;RPS27A
106	R-HSA-174495	Synthesis And Processing Of GAG, GAGPOL Polyproteins	3	15	0.012395813	0.111562318	TSG101;RPS27A
107	R-HSA-182971	EGFR downregulation	4	28	0.01256504	0.113085362	ARHGEF7;CBL;RPS27A
108	R-HSA-5689603	UCH proteinases	8	98	0.013677584	0.118154669	HIST2H2AA3;HIST2H2AA4;HIST1H2AG;TGFBR2;HIST1H2AI;HIST3H2A;HIST1H2AK;HIST1H2AM;HIST1H2AL;RPS27A
109	R-HSA-977225	Amyloid fiber formation	7	80	0.014582502	0.118154669	HIST2H3A;HIST2H2AA3;HIST1H4L;HIST2H2AA4;HIST4H4;HIST1H4K;HIST2H4A;HIST2H3C;HIST2H4B;HIST2H3D;APP;HIST1H4A;HIST1H4B;BACE1;HIST1H4E;HIST1H4F;HIST1H4C;HIST1H4D;HIST1H4I;HIST1H4J;RPS27A;HIST1H4H
110	R-HSA-5250924	B-WICH complex positively regulates rRNA expression	6	62	0.014809978	0.118154669	KAT2A;HIST2H3A;HIST2H2AA3;HIST2H2AA4;HIST1H4L;HIST4H4;HIST1H4K;HIST1H2AG;HIST2H4A;HIST2H3C;HIST2H4B;HIST2H3D;HIST1H4A;HIST1H4B;HIST1H2AI;HIST1H4E;HIST1H4F;HIST1H2AK;HIST1H4C;HIST1H4D;HIST1H4I;HIST1H2AL;HIST1H4J;HIST1H4H
111	R-HSA-212300	PRC2 methylates histones and DNA	5	45	0.014887986	0.118154669	HIST2H3A;HIST2H2AA3;HIST2H2AA4;HIST1H4L;HIST4H4;HIST1H4K;HIST1H2AG;HIST2H4A;HIST2H3C;HIST2H4B;HIST2H3D;HIST1H4A;HIST1H4B;HIST1H4E;HIST1H2AI;HIST1H4F;HIST1H4C;HIST1H2AK;HIST1H4D;HIST1H4I;HIST1H2AL;HIST1H4J;HIST1H4H
112	R-HSA-166016	Toll Like Receptor 4 (TLR4) Cascade	10	141	0.015411445	0.118154669	IRAK1;PPP2R1A;PELI1;APP;MAP3K8;MAPK9;CAP1;RPS27A
113	R-HSA-5578749	Transcriptional regulation by small RNAs	7	81	0.015497207	0.118154669	HIST2H3A;HIST2H2AA3;HIST2H2AA4;HIST1H4L;HIST4H4;HIST1H4K;HIST1H2AG;HIST2H4A;HIST2H3C;HIST2H4B;HIST2H3D

							;HIST1H4A;HIST1H4B;HIST1H2AI;HIST1H4E;AGO1;HIST1H4F;HIST1H2AK;HIST1H4C;HIST1H4D;HIST1H4I;HIST1H2AL;HIST1H4J;HIST1H4H
114	R-HSA-2262752	Cellular responses to stress	24	470	0.015500157	0.118154669	E2F1;HIST2H2AA3;HIST4H4;HIST1H4L;HIST2H2AA4;E2F3;HIST1H4K;HIST1H2AG;IL-8;HIST2H4A;HIST2H4B;MTMR3;HIST1H4A;HIST1H4B;AGO1;HIST1H4E;HIST1H4F;HIST1H4C;HIST1H4D;HIST1H4I;HIST1H4J;RPS27A;HIST1H4H;HIST2H3A;HSP27;EEF1A1;HSP90AA1;RB1;UBE2C;EHMT2;HIST2H3C;HIST2H3D;CDKN1A;VEGFA;TSC2;HIST1H2AI;MAPK9;HIST1H2AK;HIST1H2AL
115	R-HSA-427359	SIRT1 negatively regulates rRNA Expression	5	46	0.016206236	0.118154669	HIST2H3A;HIST2H2AA3;HIST2H2AA4;HIST1H4L;HIST4H4;HIST1H4K;HIST1H2AG;HIST2H4A;HIST2H3C;HIST2H4B;HIST2H3D;HIST1H4A;HIST1H4B;HIST1H4E;HIST1H2AI;HIST1H4F;HIST1H4C;HIST1H2AK;HIST1H4D;HIST1H4I;HIST1H2AL;HIST1H4J;HIST1H4H
116	R-HSA-1169410	Antiviral mechanism by IFN-stimulated genes	7	83	0.017447675	0.118154669	ARIH1;EIF4G2;EIF4G3;JAK1;RPS27A
117	R-HSA-1169408	ISG15 antiviral mechanism	7	83	0.017447675	0.118154669	ARIH1;EIF4G2;EIF4G3;JAK1;RPS27A
118	R-HSA-5654695	PI-3K cascade:FGFR2	10	144	0.017544706	0.118154669	PPP2R1A;CDKN1A;PDPK1;GAB1;TSC2;AGO1;PIK3CA;PTEN
119	R-HSA-5654710	PI-3K cascade:FGFR3	10	144	0.017544706	0.118154669	PPP2R1A;CDKN1A;PDPK1;GAB1;TSC2;AGO1;PIK3CA;PTEN
120	R-HSA-5654720	PI-3K cascade:FGFR4	10	144	0.017544706	0.118154669	PPP2R1A;CDKN1A;PDPK1;GAB1;TSC2;AGO1;PIK3CA;PTEN
121	R-HSA-5654689	PI-3K cascade:FGFR1	10	144	0.017544706	0.118154669	PPP2R1A;CDKN1A;PDPK1;GAB1;TSC2;AGO1;PIK3CA;PTEN
122	R-HSA-1250342	PI3K events in ERBB4 signaling	10	144	0.017544706	0.118154669	PPP2R1A;CDKN1A;PDPK1;GAB1;TSC2;AGO1;PIK3CA;PTEN
123	R-HSA-1257604	PIP3 activates AKT signaling	10	144	0.017544706	0.118154669	PPP2R1A;CDKN1A;PDPK1;GAB1;TSC2;AGO1;PIK3CA;PTEN
124	R-HSA-5696395	Formation of Incision Complex in GG-NER	5	47	0.017599967	0.118154669	CCL1;CETN2;RPS27A;ERCC2
125	R-HSA-212436	Generic Transcription Pathway	45	1032	0.017764204	0.118154669	E2F1;CCL1;STK11;COX7B;TAF9B;PTEN;MEN1;PDPK1;AGO1;MYC;CHD4;RPS27A;ERCC2;KAT2A;PPP2R1A;RBL2;RBL1;SMAD4;MED12;SMAD3;MED13;EHMT2;ZNF689;NOC2L;GPI;NOTCH2;CDKN1A;TNFRSF10B;MT-CO2;VEGFA;TSC2;ZIK1
126	R-HSA-5628897	TP53 Regulates Metabolic Genes	9	125	0.019217243	0.118154669	GPI;MT-CO2;COX7B;TSC2;AGO1;PTEN
127	R-HSA-450294	MAP kinase activation in TLR cascade	6	66	0.019450778	0.118154669	IRAK1;PPP2R1A;MAP3K8;MAPK9;RPS27A
128	R-HSA-180292	GAB1 signalosome	10	147	0.01988855	0.118154669	PPP2R1A;CDKN1A;PDPK1;GAB1;TSC2;AGO1;PIK3CA;PTEN
129	R-HSA-198203	PI3K/AKT activation	10	147	0.01988855	0.118154669	PPP2R1A;CDKN1A;PDPK1;GAB1;TSC2;AGO1;PIK3CA;PTEN
130	R-HSA-391160	Signal regulatory protein (SIRP) family interactions	3	18	0.019998881	0.118154669	SIRPA
131	R-HSA-5684264	MAP3K8 (TPL2)-dependent MAPK1/3 activation	3	18	0.019998881	0.118154669	MAP3K8;RPS27A
132	R-HSA-2173791	TGF-beta receptor signaling in	3	18	0.019998881	0.118154669	TGFBR2;RPS27A

		EMT (epithelial to mesenchymal transition)					
133	R-HSA-6803211	TP53 Regulates Transcription of Death Receptors and Ligands	3	18	0.019998881	0.118154669	TNFRSF10B
134	R-HSA-5625886	Activated PKN1 stimulates transcription of AR (androgen receptor) regulated genes KLK2 and KLK3	5	49	0.02062016	0.118154669	HIST2H3A;HIST2H2AA3;HIST2H2AA4;HIST1H4L;HIST4H4;HIST1H4K;HIST1H2AG;HIST2H4A;HIST2H3C;HIST2H4B;HIST2H3D;HIST1H4A;HIST1H4B;HIST1H4E;HIST1H2AI;HIST1H4F;HIST1H4C;HIST1H2AK;HIST1H4D;KDM4A;HIST1H4I;HIST1H2AL;HIST1H4J;HIST1H4H
135	R-HSA-8864260	Transcriptional regulation by the AP-2 (TFAP2) family of transcription factors	5	49	0.02062016	0.118154669	CDKN1A;VEGFA;MYC
136	R-HSA-2894862	Constitutive Signaling by NOTCH1 HD+PEST Domain Mutants	6	67	0.020749607	0.118154669	KAT2A;HDAC10;MYC;RPS27A
137	R-HSA-2644602	Signaling by NOTCH1 PEST Domain Mutants in Cancer	6	67	0.020749607	0.118154669	KAT2A;HDAC10;MYC;RPS27A
138	R-HSA-2894858	Signaling by NOTCH1 HD+PEST Domain Mutants in Cancer	6	67	0.020749607	0.118154669	KAT2A;HDAC10;MYC;RPS27A
139	R-HSA-2644606	Constitutive Signaling by NOTCH1 PEST Domain Mutants	6	67	0.020749607	0.118154669	KAT2A;HDAC10;MYC;RPS27A
140	R-HSA-2644603	Signaling by NOTCH1 in Cancer	6	67	0.020749607	0.118154669	KAT2A;HDAC10;MYC;RPS27A
141	R-HSA-75158	TRAIL signaling	2	7	0.021062515	0.118154669	TNFRSF10B
142	R-HSA-3304356	SMAD2/3 Phosphorylation Motif Mutants in Cancer	2	7	0.021062515	0.118154669	TGFBR2;SMAD3
143	R-HSA-68911	G2 Phase	2	7	0.021062515	0.118154669	E2F1;E2F3
144	R-HSA-5663202	Diseases of signal transduction	20	384	0.021455746	0.118154669	KAT2A;PPP2R1A;HSP90AA1;CBL;TGFBR2;SMAD4;HDAC10;SMAD3;PTEN;PDPK1;CDKN1A;NAPEPLD;TSC2;GAB1;DHX33;PIK3CA;MYC;RPS27A
145	R-HSA-73762	RNA Polymerase I Transcription Initiation	5	50	0.022249554	0.118154669	CCL1;KAT2A;EHMT2;CHD4;ERCC2
146	R-HSA-937061	TRIF-mediated TLR3/TLR4 signaling	8	108	0.022846186	0.118154669	IRAK1;PPP2R1A;APP;MAP3K8;MAPK9;CAP1;RPS27A
147	R-HSA-166166	MyD88-independent TLR3/TLR4 cascade	8	108	0.022846186	0.118154669	IRAK1;PPP2R1A;APP;MAP3K8;MAPK9;CAP1;RPS27A
148	R-HSA-168164	Toll Like Receptor 3 (TLR3) Cascade	8	108	0.022846186	0.118154669	IRAK1;PPP2R1A;APP;MAP3K8;MAPK9;CAP1;RPS27A
149	R-HSA-975110	TRAF6 mediated IRF7 activation in TLR7/8 or 9 signaling	3	19	0.022990319	0.118154669	IRAK1;RPS27A
150	R-HSA-5654732	Negative regulation of FGFR3	4	34	0.023630934	0.118154669	PPP2R1A;CBL;RPS27A

		signaling					
151	R-HSA-2262749	Cellular response to hypoxia	4	34	0.023630934	0.118154669	VEGFA;RPS27A
152	R-HSA-1234174	Regulation of Hypoxia-inducible Factor (HIF) by oxygen	4	34	0.023630934	0.118154669	VEGFA;RPS27A
153	R-HSA-392451	G beta:gamma signalling through PI3Kgamma	5	52	0.025753593	0.128767967	PDPK1;GNB1;PIK3CA;JAK1
154	R-HSA-5654733	Negative regulation of FGFR4 signaling	4	35	0.025905238	0.129526189	PPP2R1A;CBL;RPS27A
155	R-HSA-1266695	Interleukin-7 signaling	3	20	0.026211217	0.131056086	HIST2H3A;JAK1;HIST2H3C;HIST2H3D
156	R-HSA-380284	Loss of proteins required for interphase microtubule organizationåÊfrom the centrosome	6	71	0.026530476	0.132652382	OFD1;PPP2R1A;HSP90AA1;CETN2;TUBB4B
157	R-HSA-380259	Loss of Nlp from mitotic centrosomes	6	71	0.026530476	0.132652382	OFD1;PPP2R1A;HSP90AA1;CETN2;TUBB4B
158	R-HSA-111453	BH3-only proteins associate with and inactivate anti-apoptotic BCL-2 members	2	8	0.026951234	0.13475617	BCL2;BIM;BCL2L11
159	R-HSA-426496	Post-transcriptional silencing by small RNAs	2	8	0.026951234	0.13475617	AGO1
160	R-HSA-2559586	DNA Damage/Telomere Stress Induced Senescence	6	72	0.028126741	0.140633704	HIST2H2AA3;HIST2H2AA4;HIST1H4L;HIST4H4;HIST1H4K;HIST1H2AG;RB1;HIST2H4A;HIST2H4B;CDKN1A;HIST1H4A;HIST1H4B;HIST1H4E;HIST1H2AI;HIST1H4F;HIST1H4C;HIST1H2AK;HIST1H4D;HIST1H4I;HIST1H2AL;HIST1H4J;HIST1H4H
161	R-HSA-1980145	Signaling by NOTCH2	4	37	0.03083625	0.154181252	NOTCH2;MDK;RPS27A
162	R-HSA-445989	TAK1 activates NFkB by phosphorylation and activation of IKKs complex	4	37	0.03083625	0.154181252	IRAK1;APP;RPS27A
163	R-HSA-211000	Gene Silencing by RNA	8	115	0.03140629	0.157031452	HIST2H2AA3;HIST4H4;HIST1H4L;HIST2H2AA4;HIST1H4K;HIST1H2AG;HIST2H4A;HIST2H4B;HIST1H4A;HIST1H4B;AGO1;HIST1H4E;HIST1H4F;HIST1H4C;HIST1H4D;HIST1H4I;HIST1H4J;HIST1H4H;HIST2H3A;HSP90AA1;HIST2H3C;HIST2H3D;HIST1H2AI;HIST1H2AK;HIST1H2AL
164	R-HSA-8854518	AURKA Activation by TPX2	6	74	0.031506432	0.15753216	OFD1;PPP2R1A;HSP90AA1;CETN2;TUBB4B
165	R-HSA-2299718	Condensation of Prophase Chromosomes	5	55	0.031639996	0.158199982	HIST2H3A;HIST2H2AA3;HIST2H2AA4;HIST1H4L;HIST4H4;HIST1H4K;HIST1H2AG;RB1;HIST2H4A;HIST2H3C;HIST2H4B;HIST2H3D;HIST1H4A;HIST1H4B;HIST1H2AI;HIST1H4E;HIST1H4F;HIST1H2AK;HIST1H4C;HIST1H4D;HIST1H4I;HIST1H2AL;HIST1H4J;HIST1H4H
166	R-HSA-936964	Activation of IRF3/IRF7 mediated	3	22	0.033338234	0.16169627	CAP1;RPS27A

		by TBK1/IKK epsilon					
167	R-HSA-392851	Prostacyclin signalling through prostacyclin receptor	3	22	0.033338234	0.16169627	GNB1;GNAS
168	R-HSA-3656534	Loss of Function of TGFBR1 in Cancer	2	9	0.03341891	0.16169627	TGFBR2;SMAD3
169	R-HSA-3656532	TGFBR1 KD Mutants in Cancer	2	9	0.03341891	0.16169627	TGFBR2;SMAD3
170	R-HSA-6781823	Formation of TC-NER Pre-Incision Complex	5	56	0.033774158	0.16169627	CCL1;COPS3;RPS27A;ERCC2
171	R-HSA-397795	G-protein beta:gamma signalling	5	57	0.035995696	0.16169627	PDPK1;GNB1;PIK3CA;JAK1
172	R-HSA-532668	N-glycan trimming in the ER and Calnexin/Calreticulin cycle	5	57	0.035995696	0.16169627	GANAB;DHX33;CANX;RPS27A
173	R-HSA-5654726	Negative regulation of FGFR1 signaling	4	39	0.036283123	0.16169627	PPP2R1A;CBL;RPS27A
174	R-HSA-389356	CD28 co-stimulation	4	39	0.036283123	0.16169627	PDPK1;MAP3K8;PIK3CA;YES1
175	R-HSA-174048	APC/C:Cdc20 mediated degradation of Cyclin B	3	23	0.037241106	0.16169627	UBE2C;RPS27A
176	R-HSA-6804760	Regulation of TP53 Activity through Methylation	3	23	0.037241106	0.16169627	EHMT2;RPS27A
177	R-HSA-73887	Death Receptor Signalling	5	58	0.038305287	0.16169627	TNFRSF10B;XIAP;RPS27A
178	R-HSA-69304	Regulation of DNA replication	6	78	0.039034524	0.16169627	E2F1;E2F3;CDKN1A;RB1;RPS27A
179	R-HSA-5696394	DNA Damage Recognition in GG-NER	4	40	0.039201418	0.16169627	COPS3;CETN2;RPS27A
180	R-HSA-156902	Peptide chain elongation	7	99	0.039564444	0.16169627	RPSA;EEF1A1;RPL7;RPL21;RPS15A;RPL37;RPS27A
181	R-HSA-1253288	Downregulation of ERBB4 signaling	2	10	0.040424068	0.16169627	RPS27A
182	R-HSA-3371378	Regulation by c-FLIP	2	10	0.040424068	0.16169627	TNFRSF10B
183	R-HSA-69416	Dimerization of procaspase-8	2	10	0.040424068	0.16169627	TNFRSF10B
184	R-HSA-5625740	RHO GTPases activate PKNs	6	79	0.041080312	0.164321248	HIST2H2AA3;HIST4H4;HIST1H4L;HIST2H2AA4;HIST1H4K;HIST1H2AG;HIST2H4A;HIST2H4B;PDPK1;HIST1H4A;HIST1H4B;HIST1H4E;HIST1H4F;HIST1H4C;HIST1H4D;HIST1H4I;HIST1H4J;HIST1H4H;HIST2H3A;HIST2H3C;HIST2H3D;HIST1H2AI;HIST1H2AK;KDM4A;HIST1H2AL
185	R-HSA-175474	Assembly Of The HIV Virion	3	24	0.041367059	0.165468237	TSG101;RPS27A
186	R-HSA-5654727	Negative regulation of FGFR2 signaling	4	41	0.042249942	0.168999766	PPP2R1A;CBL;RPS27A
187	R-HSA-8868766	rRNA processing in the mitochondrion	4	41	0.042249942	0.168999766	MT-ND4;MT-ATP6;MT-CO2;MT-ND2
188	R-HSA-927802	Nonsense-Mediated Decay (NMD)	8	123	0.04361457	0.174458281	RPSA;EIF4G2;PPP2R1A;RPL7;RPL21;RPS15A;RPL37;RPS27A
189	R-HSA-975957	Nonsense Mediated Decay (NMD) enhanced by the Exon Junction	8	123	0.04361457	0.174458281	RPSA;EIF4G2;PPP2R1A;RPL7;RPL21;RPS15A;RPL37;RPS27A

		Complex (EJC)					
190	R-HSA-5633007	Regulation of TP53 Activity	10	169	0.044487088	0.177948353	PPP2R1A;PDPK1;STK11;TAF9B;EHMT2;RPS27A;CHD4;NOC2L
191	R-HSA-975956	Nonsense Mediated Decay (NMD) independent of the Exon Junction Complex (EJC)	7	102	0.045128888	0.180515552	RPSA;EIF4G2;RPL7;RPL21;RPS15A;RPL37;RPS27A
192	R-HSA-6804757	Regulation of TP53 Degradation	4	42	0.045428676	0.181714704	PPP2R1A;PDPK1;RPS27A
193	R-HSA-179409	APC-Cdc20 mediated degradation of Nek2A	3	25	0.045713055	0.182852222	UBE2C;RPS27A
194	R-HSA-450302	activated TAK1 mediates p38 MAPK activation	3	25	0.045713055	0.182852222	IRAK1;RPS27A
195	R-HSA-8852276	The role of GTSE1 in G2/M progression after G2 checkpoint	6	82	0.04761866	0.19047464	CDKN1A;HSP90AA1;RPS27A;TUBB4B
196	R-HSA-1980143	Signaling by NOTCH1	6	82	0.04761866	0.19047464	KAT2A;HDAC10;MYC;RPS27A
197	R-HSA-426486	Small interfering RNA (siRNA) biogenesis	2	11	0.047927236	0.191708944	AGO1
198	R-HSA-68689	CDC6 association with the ORC:origin complex	2	11	0.047927236	0.191708944	E2F1;E2F3
199	R-HSA-156842	Eukaryotic Translation Elongation	7	104	0.049104465	0.196417861	RPSA;EEF1A1;RPL7;RPL21;RPS15A;RPL37;RPS27A
200	R-HSA-1168372	Downstream signaling events of B Cell Receptor (BCR)	12	220	0.049302753	0.19721101	PPP2R1A;CDKN1A;PDPK1;GAB1;TSC2;AGO1;PIK3CA;PTEN;RPS27A
